# The *Plasmodium falciparum* ABC transporter ABCI3 confers parasite strain-dependent pleiotropic antimalarial drug resistance

**DOI:** 10.1016/j.chembiol.2021.06.006

**Published:** 2022-05-19

**Authors:** James M. Murithi, Ioanna Deni, Charisse Flerida A. Pasaje, John Okombo, Jessica L. Bridgford, Nina F. Gnädig, Rachel L. Edwards, Tomas Yeo, Sachel Mok, Anna Y. Burkhard, Olivia Coburn-Flynn, Eva S. Istvan, Tomoyo Sakata-Kato, Maria G. Gomez-Lorenzo, Annie N. Cowell, Kathryn J. Wicht, Claire Le Manach, Gavreel F. Kalantarov, Sumanta Dey, Maëlle Duffey, Benoît Laleu, Amanda K. Lukens, Sabine Ottilie, Manu Vanaerschot, Ilya N. Trakht, Francisco-Javier Gamo, Dyann F. Wirth, Daniel E. Goldberg, Audrey R. Odom John, Kelly Chibale, Elizabeth A. Winzeler, Jacquin C. Niles, David A. Fidock

**Affiliations:** 1 Department of Microbiology and Immunology, Columbia University Irving Medical Center, New York, NY 10032, USA; 2 Department of Biological Engineering, Massachusetts Institute of Technology, Cambridge, MA 02139, USA; 3 Division of Infectious Diseases, Allergy and Immunology, Center for Vaccine Development, St. Louis University, St. Louis, MO 63104, USA; 4 Department of Medicine, Division of Infectious Diseases, and Department of Molecular Microbiology, Washington University School of Medicine, St. Louis, MO 63110, USA; 5 Department of Immunology and Infectious Diseases, Harvard T.H. Chan School of Public Health, Boston, MA 02115, USA; 6 Infectious Disease and Microbiome Program, Broad Institute, Cambridge, MA 02142, USA; 7 Global Health Pharma Research Unit, GlaxoSmithKline, 28760 Tres Cantos, Madrid, Spain; 8 School of Medicine, University of California San Diego (UCSD), La Jolla, CA 92093, USA; 9 Drug Discovery and Development Center (H3D) and South African Medical Research Council Drug Discovery and Development Research Unit, Department of Chemistry and Institute of Infectious Diseases and Molecular Medicine, University of Cape Town, Rondebosch 7701, South Africa; 10 Division of Experimental Therapeutics, Department of Medicine, Columbia University Irving Medical Center, New York, NY 10032, USA; 11 Medicines for Malaria Venture, 1215 Geneva, Switzerland; 12 Children's Hospital of Philadelphia, Philadelphia, PA 19104, USA; 13 Division of Infectious Diseases, Department of Medicine, Columbia University Irving Medical Center, New York, NY 10032, USA

**Keywords:** *Plasmodium falciparum* malaria, ABCI3, copy-number variations, biphasic dose-response curves, cellular accumulation assays, heme fractionation, conditional knockdowns, CRISPR/Cas9, pfcrt

## Abstract

Widespread *Plasmodium falciparum* resistance to first-line antimalarials underscores the vital need to develop compounds with novel modes of action and identify new druggable targets. Here, we profile five compounds that potently inhibit *P*. *falciparum* asexual blood stages. Resistance selection studies with three carboxamide-containing compounds, confirmed by gene editing and conditional knockdowns, identify point mutations in the parasite transporter ABCI3 as the primary mediator of resistance. Selection studies with imidazopyridine or quinoline-carboxamide compounds also yield changes in ABCI3, this time through gene amplification. Imidazopyridine mode of action is attributed to inhibition of heme detoxification, as evidenced by cellular accumulation and heme fractionation assays. For the copy-number variation-selecting imidazopyridine and quinoline-carboxamide compounds, we find that resistance, manifesting as a biphasic concentration-response curve, can independently be mediated by mutations in the chloroquine resistance transporter PfCRT. These studies reveal the interconnectedness of *P*. *falciparum* transporters in overcoming drug pressure in different parasite strains.

## Highlights

• *P*. *falciparum* ABCI3 is a pleiotropic mediator of antiplasmodial drug resistance
• Strain-specific resistance was attributed to *abci3* amplification or mutant *pfcrt*
• Conditional knockdown assays identified ABCI3 as a putative drug target

## Graphical abstract

**Figure:**
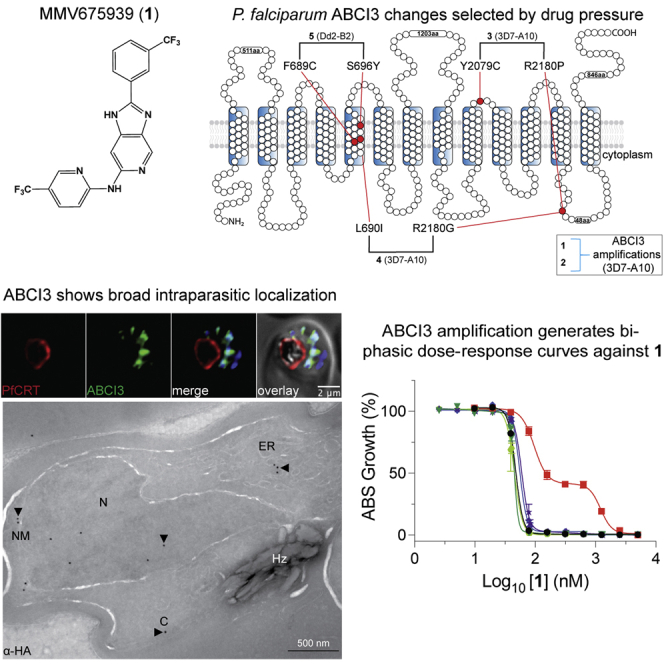

## Teaser

Murithi et al. describe ABCI3, an ATP-binding cassette transporter that conveys *Plasmodium falciparum* resistance to chemically diverse antiplasmodial compounds. *In vitro* resistance selections, gene editing, drug susceptibility, conditional knockdown, drug cellular accumulation, protein localization, and heme fractionation assays identified ABCI3 as both a resistance mediator and putative drug target.

## Introduction

An estimated 1.5 billion malaria cases and 7.6 million deaths have been averted since 2000 as a result of chemotherapy, vector control, diagnosis, and access to treatment (WHO, 2020). Despite this extraordinary success, 229 million new cases and 409,000 deaths were reported in 2019 alone (WHO, 2020), underscoring the difficult path to malaria eradication. The onset of widespread antimalarial parasite resistance, dating back to quinine resistance in 1910 and chloroquine (CQ) resistance in the 1950s (Blasco et al., 2017), has been a major obstacle in malaria drug discovery and development efforts and has continuously compromised the important role played by chemotherapy in saving lives. *Plasmodium falciparum* resistance to first-line artemisinin-based combination therapies has spread across Southeast Asia and is now threatening sub-Saharan Africa (Conrad and Rosenthal, 2019; Imwong et al., 2020; Uwimana et al., 2020). This makes it imperative that we identify new druggable targets in malaria parasites using compounds that have novel modes of antiplasmodial action.

The Malaria Drug Accelerator (MalDA) consortium is a target-guided drug-discovery platform that applies *in vitro* blood stage, liver stage, and gametocyte screening of compounds to identify novel assayable targets (Antonova-Koch et al., 2018; Cowell et al., 2018; Yang et al., 2021). Multiple whole-cell high-throughput screens have been conducted by members of the MalDA consortium and other groups to identify antiplasmodial compounds with submicromolar potencies (Antonova-Koch et al., 2018; Delves et al., 2018; Gamo et al., 2010; Guiguemde et al., 2010; Plouffe et al., 2008; Raphemot et al., 2015; Wu et al., 2015); however, the lack of target identification has stalled the development of many of these compounds into candidates for clinical application (Okombo and Chibale, 2017). We describe here a series of experiments including *in vitro* resistance selections and CRISPR/Cas9 genetic validation, drug susceptibility, conditional knockdown (cKD), drug cellular accumulation, protein localization, and heme fractionation assays to characterize culture-adapted *P*. *falciparum* resistance to five chemically distinct antiplasmodial compounds studied by the MalDA consortium. These data highlight an important role for the ATP-binding cassette (ABC) transporter ABCI3 (PF3D7_0319700) as a pleiotropic drug-resistance determinant in *P*. *falciparum*.

## Results

### Identification of point mutations or gene amplifications in ABCI3 following *in vitro* selection studies on *P*. *falciparum* asexual blood stage parasites

**Figure 1:**
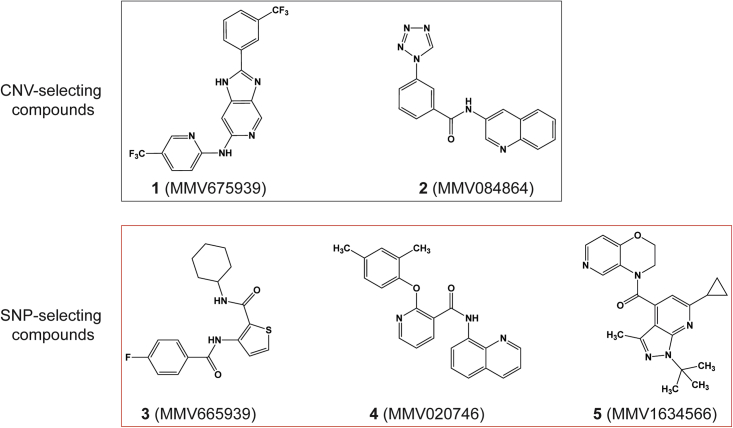
Chemical structures of MMV compounds used in this study Compounds **1** (MMV675939) and **2** (MMV084864) selected for CNVs in ABCI3 (PF3D7_0319700), whereas **3** (MMV665939), **4** (MMV020746), and **5** (MMV1634566) selected for SNPs in this gene. **1** is a 2,6-disubstituted imidazopyridine (2-(3-(trifluoromethyl)phenyl)-6-*N*-(5-(trifluoro-methyl)pyridin-2-yl)-1*H*-imidazo[4,5-*c*]pyridine); **2** is a quinoline tetrazole carboxamide (*N*-quinolin-3-yl-3-tetrazol-1-yl-benzamide); **3** is a thiophene carboxamide (*N*-cyclohexyl-3-[(4-fluoro-benzoyl)amino]-2-thiophenecarboxamide); **4** is an 8-aminoquinoline pyridine carboxamide (2-(2,4-dimethylphenoxy)-*N*-8-quinolinyl-3-pyridinecarboxamide); and **5** is a pyrazolopyridine carboxamide ((1-(*tert*-butyl)-6-cyclopropyl-3-methyl-1*H*-pyrazolo[3,4-*b*]pyridin-4-yl)-(2,3-dihydro-4*H*-pyrido[4,3-*b*][1,4]aoxazin-4-yl)methanone).

**Figure 2:**
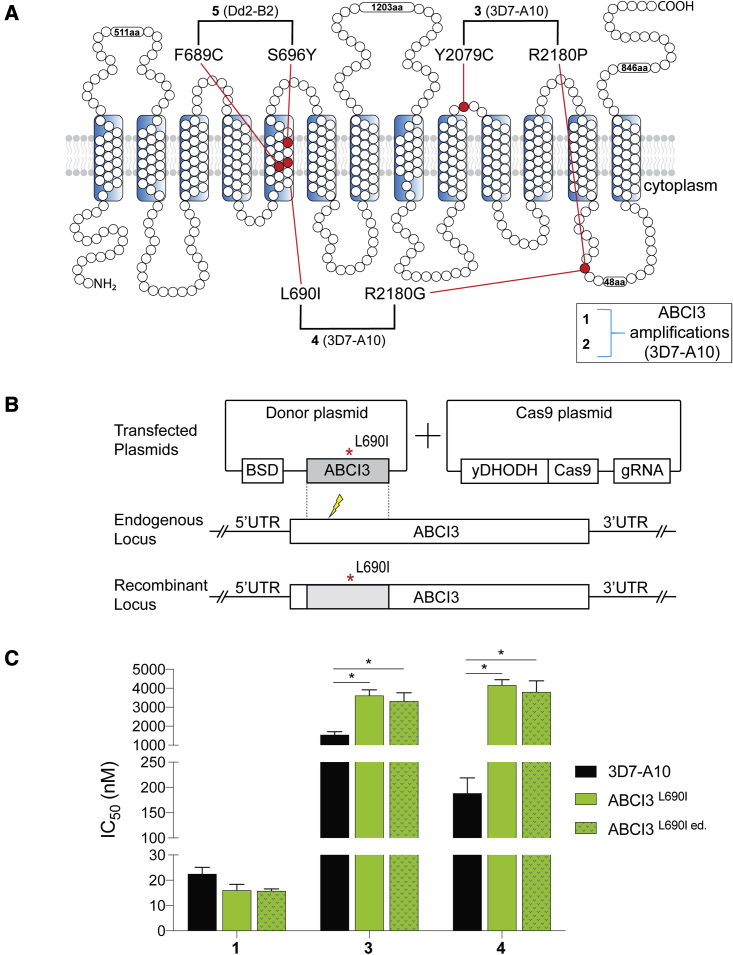
ABCI3 L690I mutation confers resistance to compounds **3** and **4** (A) Topology of ABCI3 protein based on the TMHMM, InterPro, and Uniprot structural algorithms. SNP-selecting compounds (**3–5**) generated mutations indicated in red, whereas CNV-selecting compounds are boxed. (B) The ABCI3 L690I point mutation was introduced into parental 3D7-A10 parasites using a two-plasmid CRISPR/Cas9 approach with the nearby double-stranded break site indicated with a thunderbolt. Transfected parasites were selected using blasticidin. Blasticidin-S deaminase (BSD); gRNA, guide RNA; yDHODH, yeast dihydroorotate dehydrogenase; UTR, untranslated region. (C) Parasites Cas9-edited to express the ABCI3 L690I mutation (ABCI3^L690I ed.^) phenocopied the gain of resistance observed in **4**-pressured parasites harboring this same mutation (ABCI3^L690I^). L690I conferred cross-resistance to **3** but not the CNV-selecting compound **1**. Bar graphs indicate mean ± SEM IC_50_ values of 72-h dose-response assays with asynchronous parasites. N = 5, n = 2; ∗p < 0.05, as defined using Mann-Whitney U tests of mutants versus parental 3D7-A10.

We identified possible *P*. *falciparum* resistance mechanisms to five chemically distinct compounds (Figure 1) by performing *in vitro* single-step resistance selections (Ng and Fidock, 2019). A total of 10^7^–10^9^ wild-type cloned 3D7-A10 or Dd2-B2 parasites were exposed to 3× the half-maximal growth inhibitory concentration (IC_50_) of each compound, tested in triplicate. Resistance was obtained for all five compounds, and clones were recovered by limiting dilution. Whole-genome sequencing results of these clones segregated the compounds into two distinct categories: (1) those that generated copy-number variations (CNVs) (compounds **1** and **2**); and (2) those that generated single-nucleotide polymorphisms (SNPs) (compounds **3**, **4**, and **5**) in ABCI3 (Figure 1). Specifically, compound **3** selection yielded resistant parasites harboring either the ABCI3 Y2079C or R2180P mutations, compound **4** yielded the L690I or R2180G mutations, and compound **5** yielded the F689C or S696Y mutations (Figure 2A).

Clones selected using compounds **1** and **2** all had three copies of ABCI3, compared with a single copy in the parental 3D7-A10 line, and generated biphasic dose-response curves against both compounds. These biphasic curves yielded two IC_50_ values, termed IC_50_ shifts 1 and 2. The mean ± SEM IC_50_ shifts 1 and 2 for the CNV line against compound **1** was 106 ± 8 nM and 1,249 ± 79 nM, respectively, relative to the parental IC_50_ of 47 ± 0.8 nM (Table S1). For compound **2**, mean ± SEM IC_50_ shifts 1 and 2 were 265 ± 34 nM and 4,054 ± 69, respectively, compared with the parental IC_50_ of 281 ± 19 nM.

**Figure 3:**
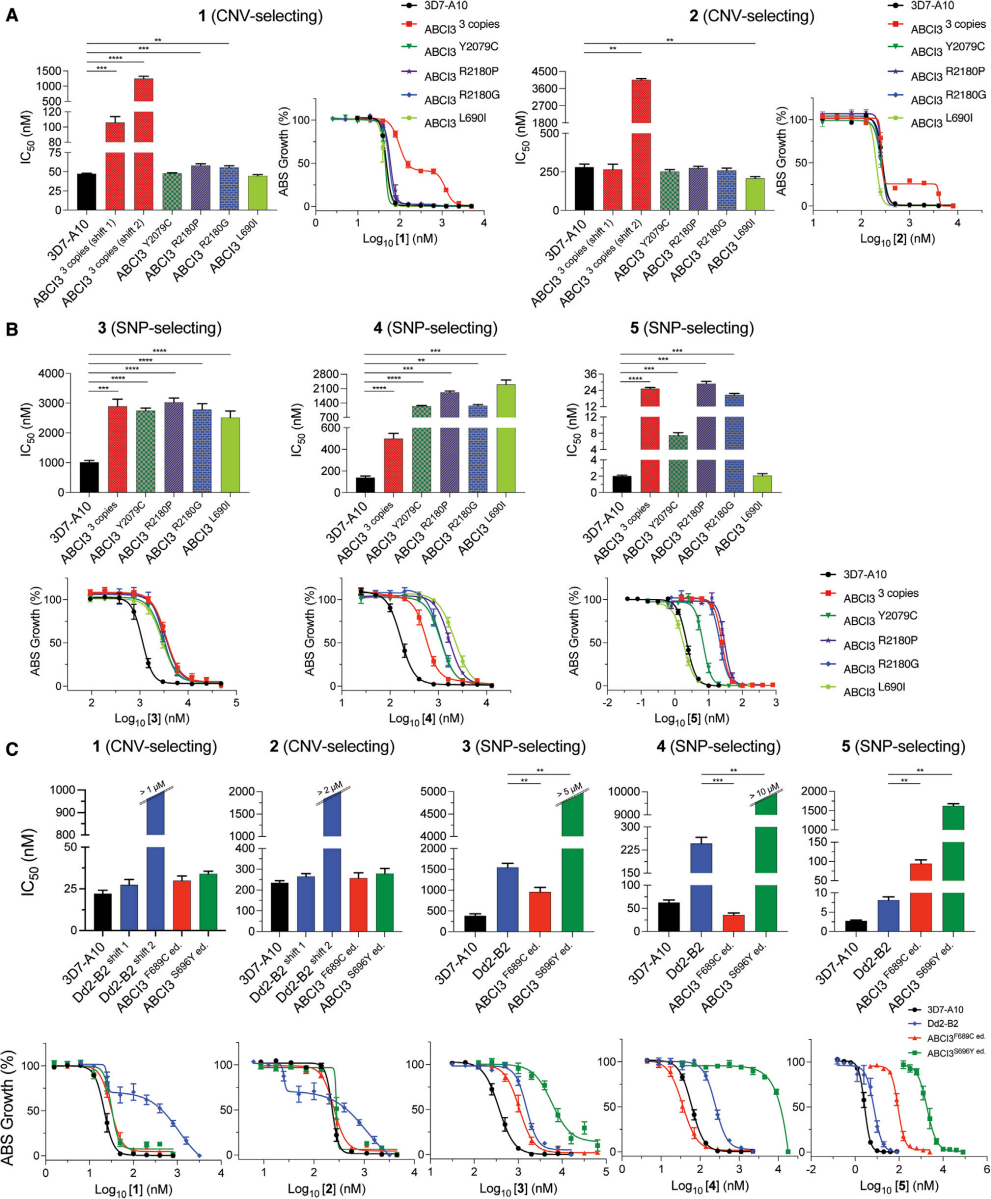
CNVs of ABCI3 confer resistance across all tested chemotypes while SNPs confer compound-specific resistance or hypersensitization (A) ABCI3 amplification in 3D7-A10 parasites mediates a biphasic gain of resistance to **1** and **2**, whose activities are unaffected by SNPs in this gene. (B) ABCI3 CNVs and most selected SNPs confer resistance in 3D7-A10 parasites to the three SNP-selecting compounds **3–5**. The L690I mutation, however, does not affect the potency of compound **5**. (C) The F689C and S696Y ABCI3 SNPs edited into Dd2 parasites eliminate the biphasic dose response observed with **1** and **2** tested against the Dd2 parent. These mutations afford compound-specific gains of resistance or hypersensitization to the SNP-selecting compounds **3–5**. Mean ± SEM IC_50_ values and dose-dependent inhibitions are shown in the bar graphs and dose-response curves, respectively, and were calculated from 72-h assays with asynchronous parasites. N ≥ 5, n = 2; ∗∗p < 0.01; ∗∗∗p < 0.001; ∗∗∗∗p < 0.0001. Mann-Whitney U tests compared resistant lines with their respective parent (3D7-A10 or Dd2-B2).

In contrast, all SNP-selecting compounds produced resistant lines with typical monophasic dose-response curves. The Y2079C and R2180P SNPs obtained from 3D7-A10 selections with compound **3** resulted in a ∼3-fold increase in IC_50_ (2,746 ± 89 nM and 3,029 ± 141 nM, respectively, compared with the parental value of 1,012 ± 64 nM). For compound **4**, the ABCI3 L690I and R2180G mutants had a ∼9- to 16-fold increase in IC_50_ (2,300 ± 217 nM and 1,268 ± 55 nM, respectively, compared with the 3D7-A10 parental value of 140 ± 14 nM). Selections using compound **5** were performed on a Dd2-B2 background, yielding ABCI3 F689C and S696Y mutations that caused a ∼11- to 180-fold increase in IC_50_, i.e., 89 ± 4 nM and 1,433 ± 24 nM compared with the parental Dd2-B2 IC_50_ of 8.0 ± 0.9 nM (Figure 3C).

To test the causal role of ABCI3 SNPs in *P*. *falciparum* resistance to these compounds, we developed a CRISPR/Cas9 gene-editing strategy to edit the L690I mutation into wild-type 3D7-A10 parasites (Figure 2B). Results with the edited (ed.) line (ABCI3^L690I ed.^) confirmed similar levels of resistance to **4** as observed with the drug-pressured line (ABCI3^L690I^) (20-fold versus 22-fold IC_50_ increases relative to the parent for edited versus selected mutants; Figure 2C and Table S3). Additionally, this mutation conferred a modest (2.0- to 2.3-fold) level of cross-resistance to another SNP-selecting compound **3** (that selected for Y2079C and R2180P). The L690I mutants showed no significant difference in susceptibility to the CNV-selecting compound **1** (Figure 2C and Table S3).

We also validated the ABCI3 F689C and S696Y mutations by introducing them into parental Dd2-B2. The edited ABCI3^F689C ed.^ and ABCI3^S696Y ed.^ lines displayed gains of resistance to **5** similar to that of the original drug-selected lines ABCI3^F689C^ and ABCI3^S696Y^ (12-fold versus 11-fold increase in IC_50_ for F689C-edited versus selected clones, and 203-fold versus 179-fold IC_50_ increase for the S696Y-edited versus selected clones, respectively; Figure 3C and Table S2). These data confirm that the L690I, F689C, and S696Y mutations in ABCI3 are drivers of parasite resistance to **4** and **5**.

We next conducted 72-h susceptibility assays using asynchronous 3D7-A10 or Dd2-B2 parental lines and their corresponding drug-resistant clones to investigate levels of resistance conferred by CNVs of ABCI3 to the SNP-selecting compounds and vice versa. Results showed that CNVs of ABCI3 conferred parasite resistance to the CNV-selecting compounds **1** and **2** and also to the three SNP-selecting compounds (∼3-fold increase in IC_50_ for compound **3**, ∼3.6-fold for compound **4**, and ∼12.5-fold for compound **5**; Table S1). In addition, the 3D7-A10-based CNV clone with three copies of ABCI3 and the Dd2-B2 parental line displayed biphasic dose-response curves when tested against **1** and **2** (Figures 3A and 3C).

We observed that the effect of mutations in ABCI3 was compound specific and sometimes sensitized parasites to other SNP-selecting compounds. For example, the ABCI3 F689C mutation conferred parasite sensitivity to **3** and **4** (∼2-fold and 7-fold decreases in IC_50_, respectively) despite conferring resistance to compound **5** (∼11-fold increase in IC_50_; Figure 3C and Table S2). With the exception of the L690I mutation that did not confer resistance to **5**, the remaining ABCI3 mutations (Y2079C, R2180P, R2180G, and S696Y) conferred resistance to the three SNP-selecting compounds (Figures 3B and 3C; Tables S1 and S2). However, none of the profiled SNPs in ABCI3 conferred resistance to **1** and **2** that had selected for ABCI3 CNVs (Figures 3A and 3C; Tables S1 and S2). Interestingly, the L690I mutant parasites (selected with **4**) showed no shift in susceptibility to **5** even though **5** selected for an adjacent ABCI3 F689C mutation (Figures 2A and 3B).

CNVs of ABCI3 only conferred a ∼2-fold increase in IC_50_ for compound **6**, which closely resembles the CNV-selecting compound **1**, when compared with parental 3D7-A10 values (Figure S1B and Table S1). In contrast, the ABCI3 CNV line had a ∼2- to 27-fold increase in IC_50_ (for shifts 1 and 2, respectively) compared with the parental line when tested with compound **1** (Table S1). 3D7-A10 selection studies with **6** did not yield resistance, despite using the same conditions as with **1**, indicating a reduced resistance liability with **6**. In a separate assay, neither SNPs nor CNVs of ABCI3 conferred resistance to a panel of seven clinical antimalarials (dihydroartemisinin, CQ, piperaquine, monodesethyl-amodiaquine, quinine, lumefantrine, and mefloquine) (Figures S1A and S1B; Table S1).

Together, these data suggest that ABCI3 constitutes a resistance pathway that is distinct from that of existing first-line drugs and provide evidence that the tested ABCI3 SNP- and CNV-selecting compounds might differ in their molecular targets. In addition, data from the SNP susceptibility assays and the cross-resistance results with compound **6** indicate that genetic changes in ABCI3 can mediate compound-specific resistance.

### Evidence that ABCI3 SNP-selecting compounds might target ABCI3

**Figure 4:**
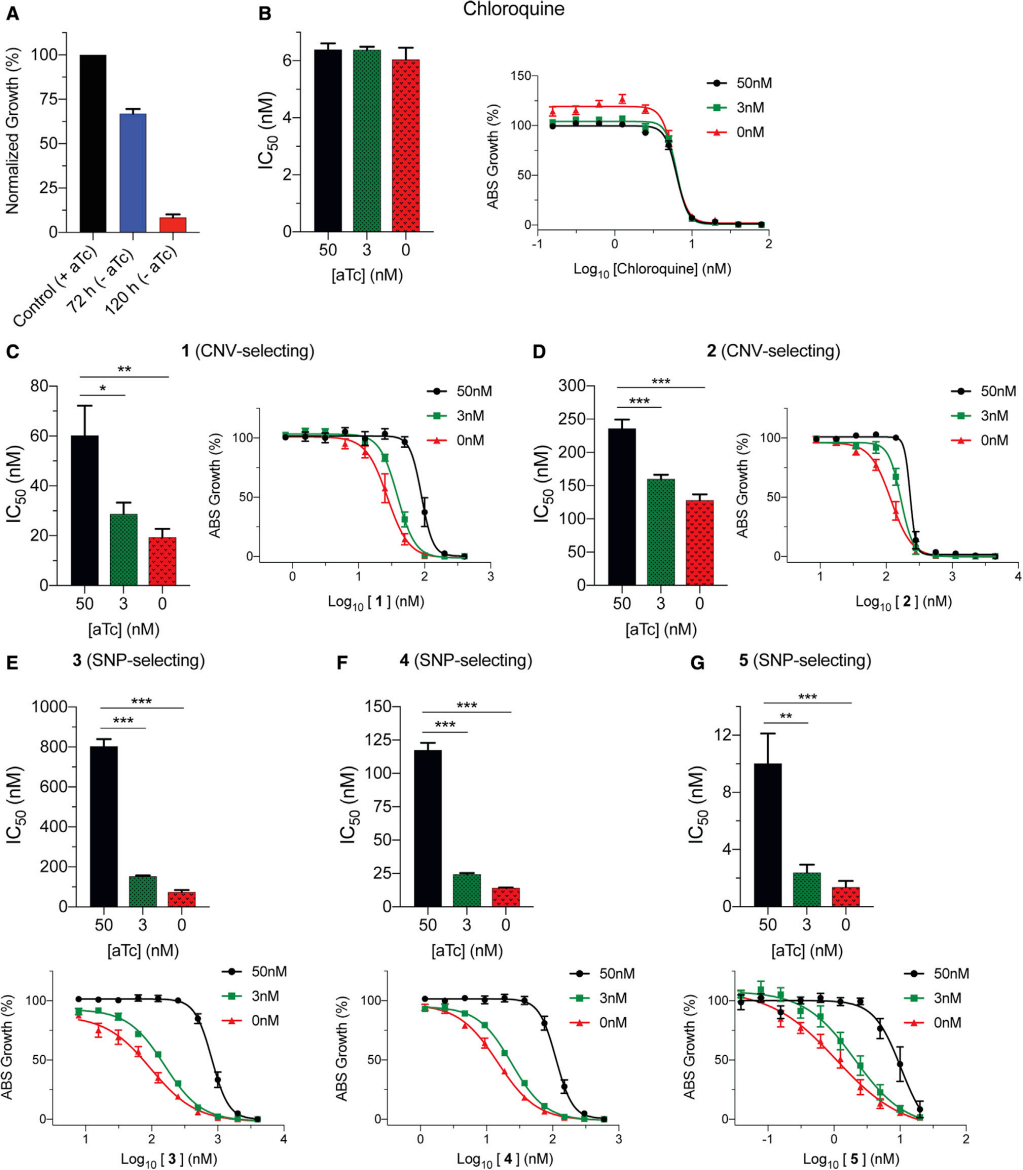
Validation of SNP-selecting compound inhibition of ABCI3 using conditional knockdown assays (A) Downregulation of ABCI3 (cKD), caused by removing aTc from the culture, reduced parasite viability by ~33% and 92% after one and two complete replication cycles, respectively, providing evidence for ABCI3 essentiality. (B) CQ does not inhibit ABCI3 and was used as a negative control. (C and D) ABCI3 cKD lines are only 2- to 3-fold sensitized to compounds **1** (C) and **2** (D). (E–G) In the absence of aTc, cKD parasites are ~7- to 11-fold sensitized to SNP-selecting compounds **3** (E), **4** (F), and **5** (G), suggesting direct inhibition of ABCI3 as a target. Bar graphs and growth curves indicate mean ± SEM IC_50_ values of 56-h dose-response assays with highly synchronized ring-stage parasites. N = 5, n = 2; ∗p < 0.05, ∗∗p < 0.01, ∗∗∗p < 0.001. Mann-Whitney U tests compared parasites with partially or fully downregulated levels of ABCI3 (achieved with 3 nM and 0 nM aTc, respectively) with parasites with wild-type ABCI3 expression (50 nM aTc).

To further explore the different interactions between ABCI3 and SNP- or CNV-selecting compounds, we engineered a cKD parasite line in which ABCI3 expression levels were regulated via the TetR-DOZI system (Ganesan et al., 2016; Nasamu et al., 2021). In this system, translation of ABCI3 protein occurs in the presence of anhydrotetracycline (aTc), but not in its absence. Wild-type ABCI3 expression levels were maintained by culturing parasites in the presence of 50 nM aTc. Medium and low ABCI3 expression levels were achieved by culturing the parasites in 3 nM and 0 nM aTc, respectively. In the absence of aTc, ABCI3 cKD parasite growth was reduced by ∼33% and ∼92% compared with control after the first and second cell cycles (72 h and 120 h, respectively; Figure 4A). We used this system to test possible inhibition of ABCI3 by conducting 56-h drug-susceptibility assays with CQ as a negative control (Figures 4B–4G and Table S4). Compound-target interactions were determined by comparing the IC_50_ of compounds against wild-type versus ABCI3 cKD parasites. We observed a modest aTc-dependent increase in parasite sensitivity to the two CNV-selecting compounds **1** and **2**, with a 2- to 3-fold increase in sensitivity at 0 nM aTc (Figures 4C and 4D; Table S4). In contrast, under the same conditions we observed a larger 7- to 11-fold increase in sensitivity to the SNP-selecting compounds **3**, **4**, and **5** (Figures 4E–4G and Table S4). cKD parasites were ∼2-fold more sensitive to compound **6** in the absence of aTc (Table S4). The observed increase in cKD sensitivity to the SNP-selecting compounds suggests a stronger inhibitory interaction with ABCI3 that is distinct from that of the CNV-selecting compounds.

### CNV-selecting compound **1** accumulates to high levels in parasites

**Figure 5:**
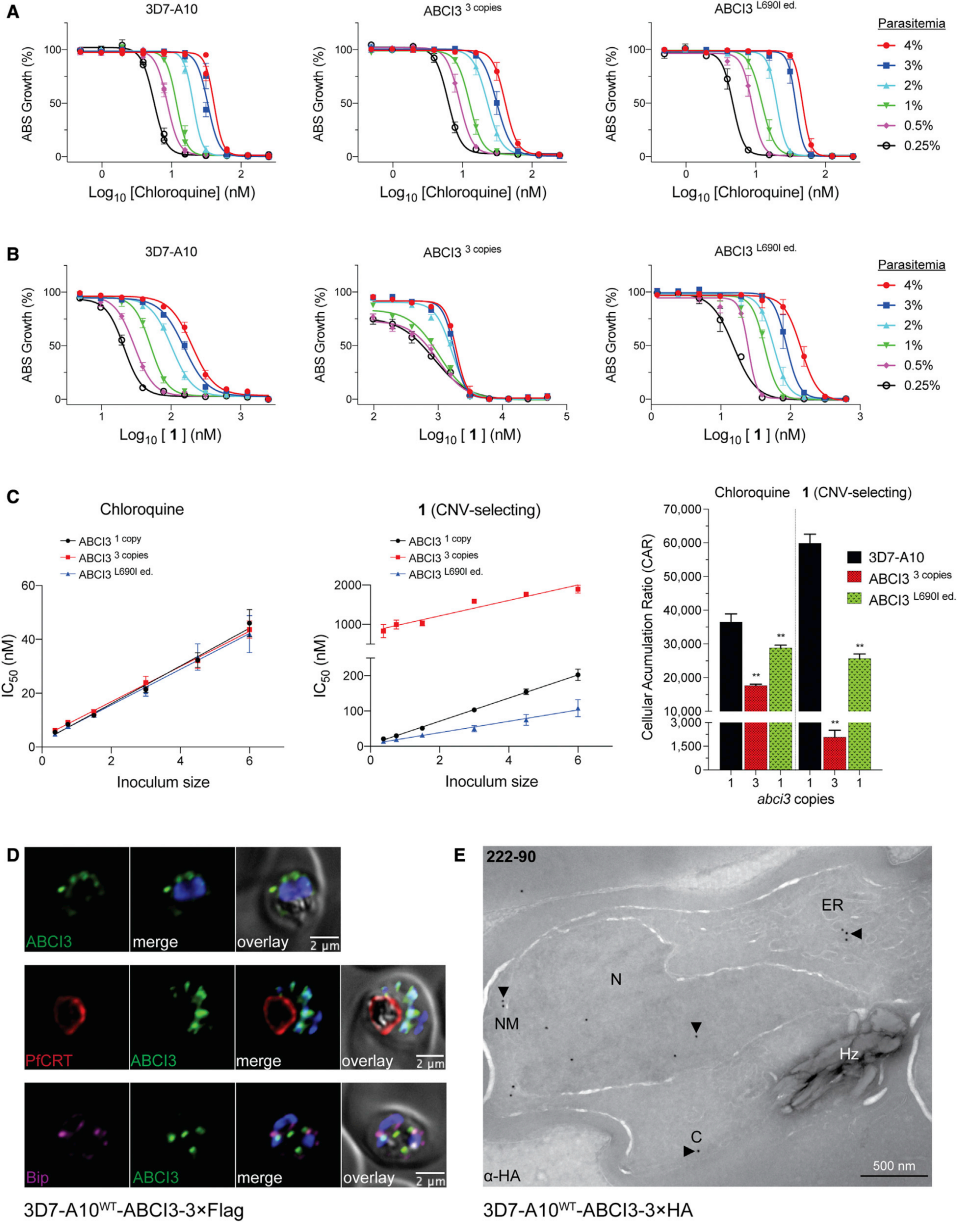
ABCI3 amplification confers resistance to **1** by potentially effluxing this compound away from its site of action (A) CQ displays an inoculum effect with a parasitemia-dependent dose-response curve unaffected by amplification or point mutation in ABCI3. Growth was determined 48 h after initiating drug treatment of highly synchronized ring-stage parasites. (B) Parental 3D7-A10 and ABCI3^L690I ed.^ parasites also display a parasitemia-dependent dose response to compound **1**. A reduced inoculum effect was observed with the ABCI3 CNV line treated with **1**. (C) The ABCI3 CNV parasite line displays ~30−fold lower cellular accumulation ratio for **1** compared with parental 3D7-A10. CQ was used as a positive control. Mean ± SEM; N = 5, n = 2; ∗∗p < 0.01. Mann-Whitney U tests versus 3D7-A10. (D) ABCI3 foci localize to punctate structures in the parasite cytosol and occasionally with the nucleus, ER, or DV (Table S8). ABCI3 FLAG-tagged parasites were stained with DAPI (4′,6-diamidino-2-phenylindole) (nucleus, blue) and antibodies specific to FLAG (green), PfCRT (DV membrane, red), and BiP (ER, magenta). Scale bars, 2 μm. (E) IEM image of an HA-tagged ABCI3 trophozoite stained with anti-HA antibodies, revealing staining in the cytosol, nucleus, nuclear membrane, and ER. Arrowheads highlight organelles of interest. C, cytosol; ER, endoplasmic reticulum; N, nucleus; NM, nuclear membrane; Hz, hemozoin crystals. Scale bar, 500 nm.

We used the parasite inoculum effect on antiplasmodial potency (Brook, 1989) to assess the cellular accumulation ratio (CAR) of one CNV- and two SNP-selecting compounds: **1**, **3**, and **4**. The CNV-selecting compound **1** displayed an inoculum-dependent IC_50_ profile similar to that of CQ against parental 3D7-A10 and L690I edited cell lines (Figures 5A and 5B). In contrast, the ABCI3 CNV line had a markedly different profile against **1**, suggesting a difference in this compound's cellular accumulation in the presence of three copies of ABCI3 (Figure 5B). We extrapolated the linear relationship between the inoculum size and the measured IC_50_ for CQ and **1** to determine the absolute IC_50_, which was then used to calculate the CAR as previously defined (Figure 5C) (Geary et al., 1990). CAR results predicted that CQ accumulated ∼1- to 2-fold more in 3D7-A10 and the L690I mutant compared with the CNV line with three copies of ABCI3, whereas compound **1** was predicted to accumulate ∼30-fold more in 3D7-A10 compared with the CNV line. Compound **1** accumulation in the L690I mutant line was estimated to be ∼2-fold less compared with parent 3D7-A10 (Figure 5C and Table S5). Compounds **3** and **4** did not display an inoculum-dependent dose response (Figure S2). These findings suggest that ABCI3 gene amplification might confer resistance to the CNV-selecting compounds by reducing their concentrations at their site(s) of antiplasmodial action. The lack of cellular accumulation for the SNP-selecting compounds suggests that they might kill parasites through modes of action that differ from those of CNV-selecting compounds.

### ABCI3 shows broad localization to multiple intraparasitic compartments

We interrogated the subcellular localization of ABCI3 by performing immunofluorescence and immunoelectron microscopy (IEM) assays on CRISPR/Cas9-edited parasites in which endogenous ABCI3 had been tagged at its C-terminal end with 3×HA (hemagglutinin) or 3×FLAG epitopes (yielding the lines 3D7-A10^WT^-ABCI3-3×FLAG or 3D7-A10^WT^-ABCI3-3×HA; Figures 5D and 5E).

Immunofluorescence assays using FLAG-specific antibodies localized ABCI3 to foci on or around the nucleus or in the parasite cytosol (Figures 5D and S3A). No substantial colocalization was observed with the digestive vacuole (DV) marker PfCRT (Kim et al., 2019), the ER marker binding immunoglobulin protein (BiP) (Witola et al., 2006), or the *cis*-Golgi marker ER lumen protein retaining receptor (ERD2) (Elmendorf and Haldar, 1993) (Figures 5D and S3A). We observed minimal association with the vesicular transport markers Rab5B and Rab7 (Stenmark, 2009) (Figure S3A). These Rab proteins are thought to contribute in part to endocytosis of host hemoglobin (Hb) and its trafficking to the DV (Elliott et al., 2008; Gnadig et al., 2020; Howe et al., 2013). IEM analysis of at least eight parasites cultured independently in triplicate (31 total images) localized ABCI3 49% of the time to the cytosol and 24% to the nucleus and nuclear membrane. Other sites of localization included the DV (8%), the plasma membrane (6%), and the ER and intracellular vesicles (13%) (Figures 5E and S3B; Table S8). Parallel processing of untagged parasites revealed no staining with these same labeling conditions. This broad intracellular distribution of ABCI3 mirrors an earlier report of mCherry-3×Myc-tagged ABCI3 localizing to intraparasitic structures and surrounding membranes (Kenthirapalan et al., 2016).

### CNV-selecting compound **1** inhibits intracellular hemozoin formation

**Figure 6:**
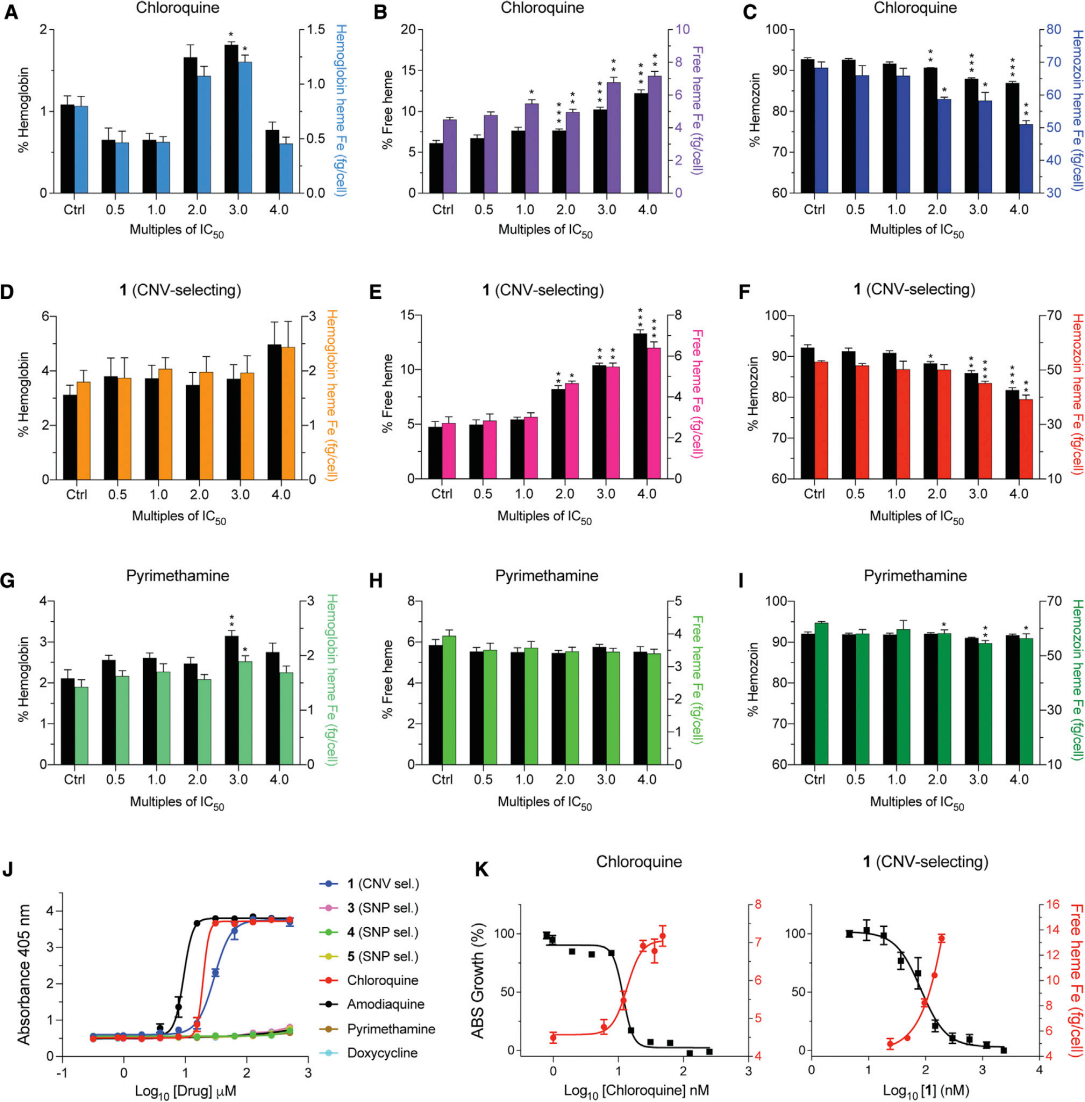
Parasites treated with compound **1** display a heme fractionation profile similar to that of CQ (A–C) Heme fractionation profile of CQ-treated NF54 parasites showing an increase in free heme and a decrease in Hz, as determined 32 h post drug exposure. (D–F) Compound **1** caused a concentration-dependent accumulation of free heme and reduction in Hz levels. (G–I) Pyrimethamine treatment did not interfere with heme or Hz accumulation. (J) Concentration-dependent inhibition of β-hematin formation by **1**, **3–5**, and four clinical antimalarial controls (N = 3 independent experiments, data shown as mean ± SEM). (K) Concentration-dependent inhibition of parasite growth obtained with CQ or **1** mirrored increasing levels of free heme, with these IC_50_ values intersecting. This result provides evidence that for **1** the inhibition of Hz formation is a primary cause of parasite growth inhibition. Percent levels of heme species are represented on the left y axis while absolute heme amounts determined from a heme standard curve and measured in femtograms per cell are represented on the right y axis. Statistical comparisons of the drug-treated lines with their untreated controls were performed using two-tailed Student's tests (with Welch's correction). ∗p < 0.05; ∗∗p < 0.01; ∗∗∗p < 0.001.

Given the evidence of its maximal activity against trophozoites (Murithi et al., 2020), partial ABCI3 localization to the DV, and the recent demonstration of inhibition of hemozoin (Hz) formation by an imidazopyridine scaffold (Horatscheck et al., 2020), we examined the potential of **1** to inhibit Hz formation in *P*. *falciparum*. As a surrogate for inhibition of heme detoxification in the parasite, we first tested the ability of this compound to inhibit the conversion of hematin to β-hematin (the synthetic equivalent of Hz) in a pyridine-based detergent-mediated assay designed to simulate the DV milieu (Carter et al., 2010). Results showed that **1** inhibited β-hematin formation (with a mean ± SEM IC_50_ of 29 ± 2.2 μM) at concentrations comparable with the 4-aminoquinoline-positive controls CQ and amodiaquine (mean ± SEM IC_50_ values: 20 ± 1.2 μM and 9 ± 1.3 μM, respectively; Figure 6J and Table S6). In contrast, SNP-selecting compounds **3–5** failed to block β-hematin formation (IC_50_ > 500 μM), similar to the negative controls pyrimethamine (an antifolate) and doxycycline (a protein synthesis inhibitor; Figure 6J and Table S6).

To further test whether **1** could target intracellular heme detoxification, we performed a cellular heme fractionation assay to test concentration-dependent effects of the compound on the three heme species: Hb, free heme (i.e., the labile form liberated by Hb proteolysis), and Hz (Combrinck et al., 2013, 2015). In this experiment, synchronized early ring-stage parasites were incubated with increasing drug concentrations, and the levels of the various heme species were quantified both as proportions of total heme extracted and as absolute amounts of heme iron (Fe) per cell (Table S6). These amounts were calculated from total quantities of Fe measured using a heme standard curve. Parasites exposed to a range of compound **1** concentrations showed concentration-dependent increases in the proportions of free heme and a corresponding decrease in Hz compared with untreated controls (Figures 6E and 6F). This profile was statistically significant at 2- to 4-fold IC_50_ concentrations and was also observed when analyzing the absolute amount of heme per cell. The mean ± SEM amount of free heme present in the untreated control was 2.7 ± 0.3 fg of heme Fe per cell while the amount present at 4-fold IC_50_ of compound **1** was 6.4 ± 0.3 fg (Table S6). This mean 2.4-fold increase in toxic free heme corresponded to a significant decrease in Hz at the equivalent IC_50_ concentration (Figure 6F) and was directly proportional to inhibition of parasite growth (Figure 6K). A similar effect was observed upon treating parasites with CQ, a known inhibitor of heme detoxification in the parasite DV (Figures 6B, 6C, and 6K). There was no concentration-related association between the amounts of free heme and Hz in parasites incubated with compounds **3–5** or with pyrimethamine, although the amount of Hz Fe appeared to decrease at higher concentrations (Figures 6H, 6I, and S4; Table S6). These decreases did not correspond to significant changes in free heme Fe levels or parasite death (Figures S4J–S4L) and might reflect a stress phenotype resulting from inhibition of other unrelated target(s). Treatment with compounds **3** and **4** caused significant increases in Hb levels, with a lesser impact on levels of free heme and Hz, suggesting potential activity of these compounds upstream in the Hb endocytosis pathway (Figures S4A, S4D, and S4G; Table S6).

### Compound **1** has a relatively high heme-binding affinity

Fe(III)PPIX can be maintained as an unaggregated and monomeric state in an aqueous solution of 40% dimethyl sulfoxide (DMSO) and is spectrophotometrically quantified by measuring the absorbance of the Soret band at 402 nm (Collier et al., 1979). We leveraged these features to investigate binding of compound **1** to monomeric Fe(III)PPIX at different pH. Drug-heme affinities were measured by titrating compounds (0–2 mM) against 10 μM aqueous Fe(III)PPIX in 40% DMSO, either at pH 7.4 (with 0.02 M HEPES) or pH 5.6 (with 0.02 MES [2-[*N*-morpholino]ethanesulfonate]) and measuring the Soret band absorbance. We then calculated the binding association constant *K* on the assumption of a 1:1 drug/heme-binding stoichiometry. Titration using CQ as a control yielded a marked decrease in absorbance, with mean ± SEM log *K* values of 5.32 ± 0.03 and 5.16 ± 0.03 at pH 7.4 and 5.6, respectively (Table S9). This agrees closely with prior studies (Dascombe et al., 2005; Egan et al., 1997). Compound **1** appeared to bind monomeric Fe(III)PPIX with moderate affinity, with mean ± SEM log *K* values of 4.00 ± 0.08 (pH 7.4) and 3.64 ± 0.03 (pH 5.6). Only weak binding associations were observed for compounds **3–5** (Table S9), consistent with their apparent inactivity in the β-hematin inhibition and cellular heme fractionation studies and providing further evidence of their separate modes of action.

### Mutant PfCRT modulates parasite susceptibility to inhibitors that select for CNVs in ABCI3

**Figure 7:**
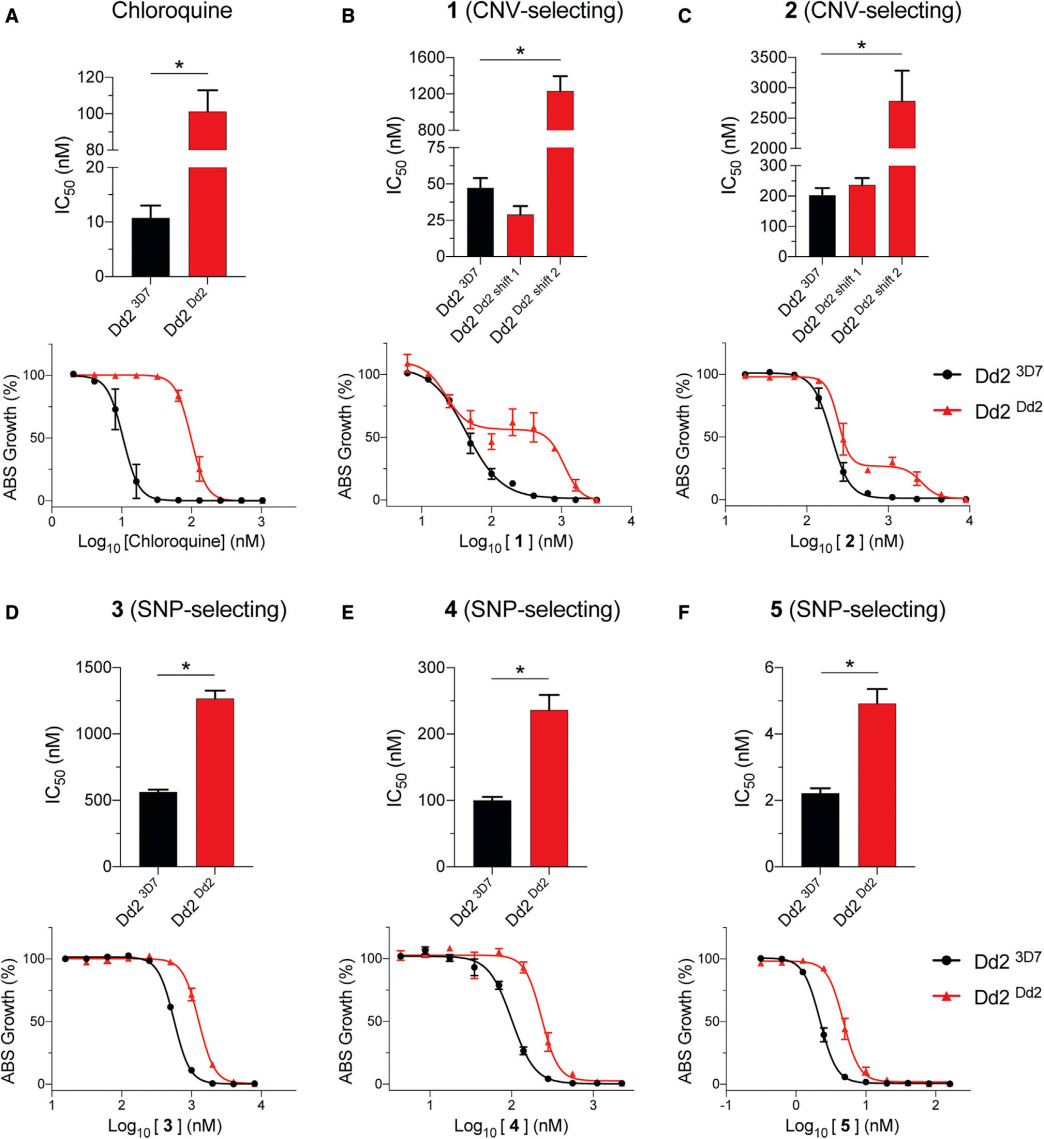
Mutant PfCRT in Dd2 parasites plays a role in susceptibility to ABCI3-associated compounds in Dd2 parasites (A) CQ resistance is conferred by the Dd2 PfCRT isoform (M74I/N75E/K76T/A220S/Q271E/N326S/I356T/R371I), showing a ~9-fold IC_50_ increase relative to isogenic gene-edited Dd2 parasites expressing the 3D7 wild-type PfCRT isoform. (B and C) The mutated PfCRT isoform confers resistance and generates biphasic dose-response curves to **1** (B) and **2** (C). (D–F) Dd2^Dd2^ PfCRT isoform confers modest (~2-fold) resistance to SNP-selecting compounds **3** (D), **4** (E), and **5** (F). Mean ± SEM; N = 4, n = 2; ∗p < 0.05. Mann-Whitney U tests compared Dd2^Dd2^ with Dd2^3D7^.

In light of the evidence that the mode of action of **1** and **2** includes inhibition of Hz formation and heme detoxification, we next assessed whether PfCRT could affect their activity, as mutations in this DV transporter can protect parasites against Hz formation inhibitors such as CQ. These assays used recombinant Dd2 parasites expressing either the mutant Dd2 PfCRT isoform that mediates CQ resistance or the wild-type CQ-sensitive 3D7 isoform (Dd2^Dd2^ and Dd2^3D7^, respectively). Dose-response assays with the control drug CQ showed the expected 9-fold higher IC_50_ and IC_90_ values in Dd2^Dd2^ parasites compared with isogenic Dd2^3D7^ parasites (Figure 7A and Table S7). Intriguingly, these isogenic lines implicated Dd2 PfCRT as a mediator of reduced parasite susceptibility to all five inhibitors linked to ABCI3 (Figures 7B–7F and Table S7).

In the case of the two CNV-selecting compounds **1** and **2**, we observed biphasic curves in Dd2^Dd2^ parasites, contrasting with a monophasic curve in Dd2^3D7^. IC_50_ values with both lines were similar, but Dd2^Dd2^ parasites showed substantially higher (14- to 26-fold) IC_90_ values (Figures 7B and 7C; Table S7). In contrast, the SNP-selecting compounds **3**, **4**, and **5** showed monophasic curves in both lines with ∼2- to 3-fold IC_50_ and IC_90_ increases in Dd2^Dd2^ parasites compared with Dd2^3D7^ (Figures 7D–7F and Table S7). This is similar to the fold IC_50_ increase obtained via mutation in ABCI3 for compound **3** but much lower than the mutant ABCI3-mediated gain of resistance to **4** and **5** (Tables S1 and S2). Assays with isogenic parasite lines expressing one or two copies of *pfmdr1*, which like *pfcrt* encodes a DV-resident multidrug-resistance transporter, showed no effect on the antiplasmodial potency of any ABCI3-associated inhibitor (Figure S5 and Table S7).

## Discussion

Our data identify ABCI3 as a pleiotropic modulator of *P*. *falciparum* asexual blood stage parasite susceptibility to a range of antiplasmodial chemotypes represented by compounds **1–5**, with resistance associated with ABCI3 amplifications or point mutations. Intriguingly, this ABC transporter appears to be broadly distributed within the parasite, mostly to punctate structures in the cytosol, with additional staining of the nucleus, nuclear membrane, DV, and plasma membrane. *In vitro* resistance selection assays using **1** and **2** generated parasites with three copies of *abci3* that conferred varying levels of resistance to all five compounds. In contrast, selections with **3–5** generated SNPs in *abci3* that did not alter parasite susceptibility to either of the two CNV-selecting compounds. None of the SNPs Y2079C and R2180P (compound **3**), L690I and R2180G (compound **4**), and F689C and S696Y (compound **5**) have been observed in *P*. *falciparum* field isolate genome datasets (reported in Databases: malariagen.net and plasmoDB.org), suggesting that these compounds have modes of action unrelated to antimalarials in clinical use.

We confirmed that ABCI3 L690I, F689C, and S696Y were the primary drivers of parasite resistance to compounds **4** and **5** using CRISPR/Cas9 gene editing. Cross-resistance studies showed that unlike CNVs, these mutations sometimes conferred no resistance or even hypersensitized parasites to other SNP-selecting compounds. The L690I and F689C mutations, while adjacent, produced distinct phenotypes when profiled against the three SNP-selecting compounds. L690I, selected with **4**, conferred resistance to both this compound and **3** but not **5**. In contrast, F689C, selected with **5**, only conferred resistance to this agent but sensitized parasites to both **3** and **4**. Strikingly, S696Y, located in the same transmembrane 5 helix as residues 689 and 690, conferred high levels of resistance (≥180-fold) to all three SNP-selecting compounds. These data provide evidence that ABCI3 SNP-mediated drug resistance is compound specific and suggest that this transporter interacts differently with SNP versus CNV-selecting compounds. These results also suggest that like the CNVs, some ABCI3 mutations can confer resistance to a broad set of chemotypes.

To explore differences in cellular accumulation between compounds and the impact of genetic changes in ABCI3, we assayed compounds **1** (CNV), **3** (Y2079C and R2180P), and **4** (L690I and R2180G) in parental 3D7-A10, ABCI3^3 copies^, and ABCI3^L690I ed.^ parasites using a previously validated inoculum effect assay (Bray et al., 1996; Brook, 1989; Geary et al., 1990; Gluzman et al., 1987; Hawley et al., 1998; Openshaw et al., 2021). These assays extrapolate linear relationships between the IC_50_ and the parasite inoculum size to quantify the CAR, defined as the ratio of the amount of drug in a parasitized red blood cell versus the amount in a similar volume of medium. Results showed that the CNV-selecting compound **1** accumulated ∼30-fold and ∼2-fold less in the ABCI3 CNV line and in the L690I mutant line, respectively, relative to the 3D7-A10 parent. The difference in CQ accumulation between the three lines was only ∼2-fold. No cellular accumulation was observed for the two SNP-selecting compounds **3** and **4**. The decreased cellular accumulation of **1** in the ABCI3^3 copies^ line suggests that ABCI3 might potentially mediate resistance by effluxing **1** from its primary site of action.

Assays measuring inhibition of Hz biomineralization from heme provide evidence that **1** interfered with the heme detoxification pathway in a CQ-like pattern, leading to the accumulation of free heme and a corresponding decrease in Hz levels. These data are consistent with recent observations of a similar effect by a 2,4-disubstituted imidazopyridine series (Horatscheck et al., 2020). In contrast, none of the tested ABCI3 SNP-selecting compounds showed any activity on the heme detoxification pathway, pointing to alternative modes of action. These findings corroborate the β-hematin inhibition results and implicate inhibition of heme detoxification by compound **1** as its likely mode of action. Recent studies have reported that the *Plasmodium* cyclic guanosine monophosphate-dependent kinase, PfPKG, might be a potential target for imidazopyridine-based derivatives (Large et al., 2019). However, our observation of optimal compound **1** activity against trophozoites (Murithi et al., 2020) does not match with the ring and schizont peak expression of PfPKG. Using a published PKG kinase assay (Baker et al., 2017), we found no detectable inhibition of recombinant PKG for any compound (tested at 10 μM for **1**, **3**, **4**, and **5**; and 0.1 μM for **2** because of limited availability), contrasting with 50% inhibition with 5 nM of the control PKG inhibitor ML10. We also observed no significant change in IC_50_ of these compounds against a previously reported PKG cKD line (Vanaerschot et al., 2020) expressing normal or low levels of this enzyme (data not shown). Falcipain 2 was also considered, as this hemoglobinase has been implicated as a target of quinoline-4-carboxamides that share some structural similarity with several of our compounds (Singh et al., 2021). Our whole-genome sequencing studies found no evidence for a role of falcipain 2 herein.

Our observation of an unusual biphasic dose-response curves with **1** and **2**, tested in growth-inhibition assays against the 3D7-based ABCI3 CNV parasite line (selected with **1**) and the non-drug-pressured Dd2-B2 line recalls the biphasic responses to piperaquine (PPQ) seen in PPQ-resistant parasites (Bopp et al., 2018; Duru et al., 2015; Ross et al., 2018). These biphasic relationships have been attributed to either the presence of multiple parasite stages that differ in their susceptibility, polypharmacology with multiple modes of actions, concentration-dependent off-target activity (which can sometimes be overcome with subtle chemical changes), or concentration-dependent activation of drug efflux mechanisms (Co et al., 2009; Le Manach et al., 2018; Patel et al., 2008; Wicht et al., 2020).

For **1** and **2**, we also identified mutant PfCRT as a contributor to susceptibility in Dd2 parasites. Growth-inhibition assays with these compounds revealed biphasic dose-response curves in CQ-resistant Dd2^Dd2^ parasites expressing Dd2 mutant *pfcrt*, as opposed to a monophasic profile obtained with isogenic CQ-sensitive Dd2^3D7^ parasites expressing 3D7 wild-type *pfcrt*. This shift to a biphasic curve in the edited *pfcrt*-mutant line mirrors the observation with non-edited Dd2, suggesting that mutant *pfcrt* is the major driver of this biphasic response in Dd2 parasites (which harbor a sole copy of *abci3*), as opposed to 3D7 that shows a classic monophasic curve. Of note, *pfcrt*-edited Dd2^Dd2^ differs from Dd2^3D7^ by eight amino acid substitutions in this transporter (M74I/N75E/K76T/A220S/Q271E/N326S/I356T/R371I), which collectively mediate resistance via a gain of CQ transport (Kim et al., 2019; Riegel and Roepe, 2020; Shafik et al., 2020). These data suggest that the biphasic gain of resistance observed with Dd2 PfCRT might reflect its ability to transport **1** and **2**. A similar biphasic dose-response profile was observed earlier with the PfCRT variants Dd2+F145I and Dd2+T93S that confer PPQ resistance, also via a gain of transport (Dhingra et al., 2019b; Kim et al., 2019).

Both CQ and PPQ accumulate by up to several-thousand-fold in drug-sensitive parasites, driven presumably by their gain of protonation (to PPQ^4+^ or CQ^2+^) in the highly acidic DV and their binding to Hz, combined with the absence of an appropriately mutated PfCRT transporter that can efflux these drugs back into the parasite cytosol (Bray et al., 1998; Kim et al., 2019; Wicht et al., 2020). Our studies revealed even higher levels of compound **1** accumulation in drug-sensitive 3D7 parasites compared with CQ, and **1** and **2** both harbor multiple sites for protonation. Conceivably, these two ABCI3 CNV-selecting compounds might act primarily in the DV, with the CQ-resistant Dd2 PfCRT isoform able to efflux them into the parasite cytosol. This proposed mode of action is consistent with our observation that **1** inhibited Hz formation and parasite growth at equivalent IC_50_ values.

Selection studies with **1** and **2** in initially fully sensitive 3D7 parasites revealed a different path to resistance. This line harbors wild-type PfCRT that transports far less CQ than the Dd2 isoform and presumably also transports little or no **1** and **2**. Selection with these compounds resulted in ABCI3 amplification, which we hypothesize results in their lack of accumulation in the DV. Indeed, we observed significantly less cellular accumulation of **1** in 3D7 parasites that had acquired three copies of *abci3*. This transporter was observed mostly in the parasite cytosol, presumably associated with membrane-bound structures, and to only a minor extent with the parasite DV. These data suggest that multicopy ABCI3 might be able to efflux compound out of the parasite or perhaps, as a result of increased levels on the DV, might enable efflux from this site of drug action. Interestingly, *abci3* amplification in 3D7 parasites created a biphasic dose-response curve, phenocopying the dose response obtained via mutant PfCRT in Dd2 parasites. To our knowledge, this is the first instance whereby the resistance determinant to a particular compound with antiplasmodial activity differs depending on the parasite genetic background.

Our studies point to a separate mode of action of compounds **3–5** compared with **1** and **2**, based on their differences in dose response, cellular accumulation, heme fractionation, and genetic changes causing resistance. These results collectively suggest that **3–5** act outside the DV. Intriguingly, Dd2 mutants resistant to **5** (harboring the ABCI3 mutations F689C or S696Y) lost their biphasic dose response to **1** and **2**, providing evidence that these SNPs reversed mutant PfCRT-mediated resistance. Results with our ABCI3 cKD lines (generated in NF54, the parent of 3D7) were informative in showing that reduced levels of ABCI3 caused increased parasite sensitivity to all five tested compounds. This susceptibility profile, however, differed markedly between the SNP-selecting (**3**, **4**, and **5**) and CNV-selecting (**1** and **2**) compounds. While the cKD parasites were ∼7- to 11-fold more sensitive to **3**–**5** in the absence of aTc, they were only ∼2- to 3-fold more sensitive to **1** and **2**. We conjecture that ABCI3 itself might be a target of **3–5**. This distinction between the modes of action between the SNP- and CNV-selecting compounds is consistent with our recent observations of differences in their timing of action, with **1** showing a peak of activity in trophozoites (the stage of maximal heme detoxification) versus **3** and **4** that showed cumulative activity across all stages (Murithi et al., 2020). ABCI3 was also observed in proteomic studies to be abundant throughout all asexual blood stages (Pease et al., 2013). For compounds **3–5**, Dd2 PfCRT caused a minor (2-fold) increase in the IC_50_. These data suggest an intricate connection between PfCRT and ABCI3 in dictating parasite susceptibility to these compounds. We note that both transporters are apparently essential for asexual blood stage parasite growth, as previously demonstrated for PfCRT and evidenced herein with our cKD studies that showed a loss of growth upon ABCI3 depletion (Kenthirapalan et al., 2016; Waller et al., 2003; Zhang et al., 2018). In contrast, we found no evidence for a role of *pfmdr1* amplification, which can modulate parasite susceptibility to the first-line drugs lumefantrine and mefloquine and which, like *pfcrt*, encodes a DV-resident transporter (Wicht et al., 2020).

ABCI3 belongs to the AAA+ superfamily of ATPases found in all kingdoms of living organisms, where they participate in diverse cellular processes including membrane fusion, proteolysis, and DNA replication. Other potential functions for members of this superfamily include protein folding and unfolding, assembly or disassembly of protein complexes, protein transport and degradation, replication, recombination, repair, and transcription (Borst, 2020; Thomas and Tampe, 2020). More research, including solving the structure of the protein and solute and drug-transport assays, is required to determine whether some of these potential endogenous functions apply to ABCI3 and to better understand how this function is co-opted through amplification or point mutations to mediate antiplasmodial drug resistance.

## Significance

**The persistent threat of multidrug resistance mediated by *Plasmodium falciparum* transporters makes it imperative to identify their interactions with first-line drugs and antiplasmodial compounds in the discovery and development pipeline. Here we report on *P*. *falciparum* ABCI3, an ATP-binding cassette transporter with broad cellular localization that can confer antiplasmodial drug resistance through gene amplifications or point mutations. Results from *in vitro* selections, validated through gene editing, conditional knockdown, and cellular accumulation studies, revealed that ABCI3 is the primary target of point mutation-selecting carboxamide-containing compounds** 3–5**. We also observed that the gene amplification-selecting imidazopyridine-containing compound** 1 **targets the heme detoxification pathway. These findings support the hypothesis that although ABCI3 is a resistance mediator to both SNP- and CNV-generating compounds, the latter have a separate mode of action.**

**The unusual biphasic dose-response curves observed with compounds** 1 **and** 2 **against a 3D7-A10-based ABCI3 CNV line and a Dd2-B2 line revealed intriguing insights into parasite biology. ABCI3 amplifications in 3D7-A10 resulted in decreased intracellular accumulation of compound** 1**, presumably via drug being effluxed away from its site of action. Growth-inhibition data for** 1 **and** 2 **assayed against isogenic Dd2**^**Dd2**^ **(CQ-resistant) and Dd2**^**3D7**^ **(CQ-sensitive) lines suggested a different mode of parasite resistance in Dd2-B2 parasites with the CQ-resistant Dd2 PfCRT isoform able to confer resistance, also presumably through gain of transport properties resulting from PfCRT mutations. Intriguingly, point mutations in ABCI3 neutralized this PfCRT-driven resistance.**

**This study highlights unique strain-dependent ways in which *P*. *falciparum* is able to evade antiplasmodial drug action. The study also identifies ABCI3 as a pleiotropic drug transporter to consider when assessing the risk of resistance to new antimalarials arising in the discovery and development pipeline.**

## STAR★Methods

### Key resources table

**Table:** 

REAGENT or RESOURCE	SOURCE	IDENTIFIER
**Antibodies**		

Mouse monoclonal anti-HA	Sigma-Aldrich	Cat# H3663; RRID: AB_262051
Goat anti-mouse IgG (H+L)	Thermo Fisher Scientific	Cat# A-11029; RRID: AB_2534088
Anti-rabbit IgG, HRP-linked	Cell Signaling Technology	Cat# 7074; RRID: AB_2099233
Rabbit anti-Flag epitope	Genscript	Cat# A01868
Mouse anti-Flag epitope	Genscript	Cat# A00187; RRID: AB_1720813
Rabbit anti-ERD2	BEI Resources	Cat# MRA-1
Rabbit anti-BiP	Min Zhang	N/A
Rat anti-Rab5B	Gordon Langsley	N/A
Rat anti-Rab7	Gordon Langsley	N/A
Mouse monoclonal anti-PfCRT	Ilya Trakht	N/A
Goat anti-mouse IgG1	Thermo Fisher	Cat# A-21121; RRID: AB_2535764
Goat anti-rabbit IgG (H+L)	Thermo Fisher	Cat# A32732; RRID: AB_2633281
Goat anti-rabbit IgG (H+L)	Jackson ImmunoResearch Laboratories	Cat# 111-005-003; RRID: AB_2337913

**Biological Samples**		

See below (cell lines)		

**Chemicals**, **Peptides**, **and Recombinant Proteins**		

All tested antimalarials and their structures are available in Figures 1 and S1.		
MMV675939	H3D, University of Cape Town, South Africa	N/A
MMV665939	Medicines for Malaria Venture, Geneva, Switzerland	N/A
MMV020746	Medicines for Malaria Venture, Geneva, Switzerland	N/A
MMV084864	Medicines for Malaria Venture, Geneva, Switzerland	N/A
MMV1634566	Medicines for Malaria Venture, Geneva, Switzerland	N/A
MMV675097	H3D, University of Cape Town, South Africa	N/A
SYBR Green	Thermo Scientific	Cat# S7563
MitoTracker Deep Red	Thermo Scientific	Cat# M22426
Renilla-Glo(R) Luciferase Assay System	Promega	Cat# E2750
Amaxa nucleofector solution 2	Lonza	Cat# V4XP-3024
Anhydrotetracycline	Sigma-Aldrich	Cat# 37919
WR99210	Jacobus Pharmaceuticals	N/A

**Critical Commercial Assays**		

Renilla-Glo(R) Luciferase Assay System	Promega	Cat# E2750

**Experimental Models**: **Cell Lines**		

*P*. *falciparum* line 3D7-A10	Goldberg Lab, Washington University, St. Louis, MO, USA	3D7-A10 clone
*P*. *falciparum* line Dd2-B2	Wellems Lab, NIAID, Rockville, MD, USA	Dd2-B2 clone
*P*. *falciparum* line NF54	Wellems Lab	NF54
*P*. *falciparum* line NF54^pCRISPR^	Niles Lab, MIT, Cambridge, MA, USA	NF54^pCRISPR^
*P*. *falciparum* lines Dd2 ^3D7^ and Dd2 ^Dd2^	Fidock Lab, Columbia University Medical Center, New York, NY, USA	*pfcrt*-modified Dd2 ^3D7^ and Dd2 ^Dd2^ clones

**Oligonucleotides**		

(**see**Table S10)		N/A

**Recombinant DNA**		

pSN054 vector	(Nasamu et al., 2021)	NA
3D7-A10WT-ABCI3-3×Flag	Fidock Lab	NA
3D7-A10WT-ABCI3-3×HA	Fidock Lab	NA

**Software and Algorithms**		

GraphPad Prism Version 8	GraphPad Software, San Diego, CA, USA	www.graphpad.com
AMT Image Capture Engine V602 software	Advanced Microscopy Techniques	www.amtimaging.com

N/A, not applicable.

### Resource availability

#### Lead contact

Further information and requests for resources and reagents should be directed to the lead contact, David Fidock (df2260@cumc.columbia.edu).

#### Materials availability

Please note that amounts of experimental compounds may be restricted and might require resynthesis. Chemical structures for the compounds used in these studies are shown in Figures 1 and S1B.

### Experimental model and subject details

Asexual blood stage *P*. *falciparum* parasites were cultured at 3% hematocrit in O^+^ human erythrocytes in RPMI-1640 medium supplemented with 50 μM hypoxanthine, 2.1 g/L NaHCO_3_, 2 mM L-glutamine, 25 mM HEPES, 0.5% (w/v) AlbuMAXII (Invitrogen) and 10 μg/mL gentamycin at 37°C in flasks gassed with 5% CO_2_/5% O_2_/90% N_2_. The 3D7-A10, Dd2-B2 and NF54 *P*. *falciparum* parasite lines have been previously reported (Murithi et al., 2020; Ponnudurai et al., 1982). 3D7-A10 and Dd2-B2 are clones of the parasite lines 3D7 and Dd2, respectively, and NF54 is the parental isolate from which 3D7 was earlier cloned. Dd2 ^Dd2^ and Dd2 ^3D7^ isogenic *pfcrt*-edited lines were reported in (Dhingra et al., 2019a). The human erythrocytes were sourced ethically from blood banks with anonymized blood donors and their research use for cell culture was in accordance with terms of informed consent under an IRB/EC approved protocol.

### Method details

#### Compounds, resistance selections and *in vitro* drug susceptibility assays

Compounds **2-5** were kindly provided by the Medicines for Malaria Venture (Geneva, Switzerland), and **1** and **6** were synthesized at the Drug Discovery and Development Centre (H3D) at the University of Cape Town in South Africa as part of the SoftFocusKinase 59 (SFK59) library (Le Manach et al., 2018; Nchinda et al., 2018). Parasites resistant to **1** were obtained from single-step selections where 10^7^ 3D7-A10 parasites in triplicate were cultured with a continuous 3×IC_50_ drug pressure. For the SNP-selecting **3** and **4**, single-step selections were run on 10^9^ 3D7-A10 parasites. The same parasite numbers and methods were used for Dd2-B2 selections using compound **5**. Resistant clones were obtained from bulk cultures by limiting dilution. For the susceptibility experiments, compounds were assayed using 2-fold dilutions with inhibition measured after 72 h. Parasite viability was determined by staining the parasites with SYBR Green and MitoTracker Deep Red (Life Technologies) followed by flow cytometry (Accuri C6, BD Biosciences or iQue Plus, Sartorius) (Ekland et al., 2011). IC_50_ values were derived by nonlinear regression (Prism 7, GraphPad).

#### Whole-genome sequencing analysis

Sequencing of *P*. *falciparum* clones resistant to compounds **2-4** was performed by the Winzeler lab, using methodology reported in (Fisher et al., 2020) and described below. For **1** and **5**, resistant clones were sequenced by the Fidock lab, using methods reported in (Dorjsuren et al., 2021; Vanaerschot et al., 2020) and described below. Paired-end libraries were sequenced on Illumina HiSeq or MiSeq instruments.

In the Winzeler lab, the Nextera XT kit (Illumina) was used to prepare DNA libraries from samples for whole-genome sequencing using the dual index protocol (Fisher et al., 2020). The libraries were run on an Illumina HiSeq 2500 in rapid run mode with 100-bp paired-end reads. Reads were aligned to the *P*. *falciparum* 3D7 reference genome (PlasmoDB v. 13.0) as described previously (Manary et al., 2014). Single nucleotide polymorphisms (SNPs) and indels were called with the Genome Analysis Toolkit’s (GATK) HaplotypeCaller. Variants were filtered by quality scores and sequencing bias statistics based on GATK’s default filtering parameters. SNPs were filtered out if they met any of the following criteria: quality depth (QD), <2.0; mapping quality (MQ), <50.0, Phred-scaled P value using Fisher’s exact test to detect strand bias (FS), >60.0; symmetric odds ratio (SOR), >4.0; Z-score from Wilcoxon rank sum test of alternative versus reference read mapping qualities (MQRankSum), less than 12.5; ReadPosRankSum (RPRS) parameter, less than 8.0. Indels were filtered out if they met any of the following criteria: QD, <2.0; RPRS, less than 20.0; FS, >200.0. Variants were annotated using snpeff (version 4.2). Custom scripts were used to compare the variants between the parent sequence and the resistant clones.

In the Fidock lab, whole-genome sequencing of genomic DNA from parental and resistant clones employed an Illumina TruSeq DNA PCR-Free library preparation protocol and a MiSeq sequencing platform. Briefly, 2 μg of genomic DNA were sheared to a mean length of 550 bp, end-repaired, adenylated on their 3’ ends and ligated to indexed adaptors. Samples were pooled and sequenced on Illumina MiSeq flow cells to obtain 300 bp paired-end reads. Sequence data were aligned to the *P*. *falciparum* 3D7 genome (PlasmoDB version 48) using BWA (Burrow-Wheeler Alignment). We used Samtools and Picard to remove PCR duplicates and reads that did not map to the reference genome. Reads were realigned around indels using GATK RealignerTargetCreator and base quality scores were recalibrated using GATK Table-Recalibration. GATK HaplotypeCaller (version 4.1.8; Min Base quality score ≥ 18) was used with the clones to identify all possible variants, which were filtered based on quality scores (variant quality as function of depth QD > 1.5, mapping quality > 40) and read depth (depth of read > 5) to obtain high-quality SNPs. These SNPs were annotated using snpEFF. The list of variants from the resistant clones were compared against the 3D7-A10 parent to obtain homozygous SNPs that were present exclusively in the resistant clones. IGV was used to confirm the SNPs present in the resistant clones. BicSeq was used to discover copy number variants (CNVs) against the 3D7-A10.

#### Genome editing

The ABCI3 L690I mutation was introduced into 3D7-A10 parasites using a two-plasmid CRISPR/Cas9 system. Cas9 was derived from *Streptococcus pyogenes* and was fused to the selectable marker yDHODH that confers resistance to DSM1 (Gujjar et al., 2009). Both Cas9 and the selectable marker were expressed from the *P*. *falciparum* calmodulin promoter. The Cas9 plasmid also contained a guide RNA expressed from the U6 promoter. Guide RNA sequences were selected using ChopChop, an online gRNA design tool, and were based on their proximity to the mutation of interest, GC content, and absence of poly A/T tracks (http://chopchop.cbu.uib.no). The donor plasmid contained the *abci3* fragment with the L690I mutation and blasticidin-S deaminase (*bsd*), a selectable marker that protects against blasticidin (Sigma-Aldrich). Plasmid transfections were conducted using an Amaxa nucleofector (Janse et al., 2006). Briefly, a cell pellet of 7×10^8^ highly synchronized and magnet-purified 3D7-A10 mature schizonts were first re-suspended in 100 μl of Nucleofector Solution 2 (with the supplement added) that had been pre-warmed to room temperature. This was mixed with 10 μg of plasmid DNA in a volume of 5 μl deionized water in a cuvette and electroporated using the Amaxa U-033 program. Parasites were allowed to re-invade fresh RBCs in complete media pre-warmed to 37^o^C. Transfected parasites were selected with 2 μg/ml blasticidin for six days starting the day after electroporation, and parasites were maintained thereafter in complete media until recrudescence. Gene editing was assessed via Sanger sequencing of PCR products amplified from bulk cultures. Edited parasite clones were obtained by limiting dilution.

Edited parasites were assayed for drug susceptibility by flow cytometry (Ekland et al., 2011). Briefly, we exposed predominantly ring-stage parasite cultures at 0.2% parasitemia, 1% hematocrit and 200 μl volume per well (in 96-well flat-bottomed plates) for 72 hr to a range of ten drug concentrations that were 2-fold serially diluted in duplicates along with drug-free controls. Drug dilutions were inoculated using a Tecan Evo100 liquid handler. After 72 hr, 5 μl aliquots of each well were transferred into a new 96-well round-bottomed plate and stained for 30 min at 37°C with 40 ml of a mixture of 1× SYBR Green and 2× MitoTracker Deep Red (Thermo Scientific) as nuclear and vital dyes, respectively. Parasitemias were assessed by flow cytometry on an iQue Plus (Sartorius). Flow counts were analyzed using FlowJo software (FlowJo LLC). Percent parasite survival (normalized to 100%) was plotted against log drug concentrations and non-linear regression was used to determine IC_50_ values (GraphPad Prism version 9).

C-terminal 3×Flag or 3×HA tagging of ABCI3 was achieved by transfecting highly sorbitol-synchronized 3D7-A10 ring-stage parasites with an all-in-one CRISPR/Cas9 plasmid. The *P*. *falciparum* codon-optimized Cas9 endonuclease was derived from *Streptococcus pyogenes* and was expressed under a calmodulin promoter. The plasmid also carried a human DHFR (hDHFR) selectable marker (that confers resistance to WR99210) under a *P*. *chabaudi dhft-ts* promoter and the guide RNA (gRNA) sequence under a U6 promoter. Guide RNAs were selected using ChopChop 9 (https://chopchop.cbu.uib.no). 10^8^ parasites were electroporated with purified circular plasmid DNA as described (Fidock et al., 1998). Briefly, a 5 mL culture of 3D7-A10 (≥ 10% rings) was washed and resuspended in 220 μL 1× Cytomix. This mixture was then added to 50 μg of plasmid DNA and electroporated at a voltage of 0.31 kV and capacitance of 950 μF using a Gene-Pulser (Bio-Rad) (Adjalley et al., 2010). Starting on the day after the transfections, the cultures were maintained in 2.5 nM WR99210 until recrudescence (Fidock and Wellems, 1997). Successful gene editing was assessed via Sanger sequencing of PCR products amplified from bulk cultures. Edited parasite clones were obtained by limiting dilution. Successful gene tagging was confirmed via PCR, Sanger sequencing, immunofluorescence, and immuno-EM assays. Oligonucleotide primers used in this study are listed in the Key Resources table.

#### Generation of conditional knockdown parasite lines

We utilized CRISPR-Cas9 to modify the native PfABCI3 (PF3D7_0319700) locus by inserting the linearized pSN054 donor vector (Nasamu et al., 2021), which incorporates a 10× aptamer array and the TetR-DOZI expression cassette containing the blasticidin S-deaminase gene, the reporter gene Renilla luciferase (RLuc), and the TetR-DOZI fusion protein (Ganesan et al., 2016). The right homology region (264 bp) was PCR amplified and inserted into the pSN054 vector using the I-SceI restriction site. Fragments corresponding to the left homology region (426 bp) fused to the re-codonized 3'-end of the gene (bp 9991-10092) without the stop codon as well as the target-specifying guide RNA sequence were synthesized using the BioXP™ 3200 System (SGI-DNA) and cloned into pSN054 using the restriction sites FseI/AsisI and AflII, respectively. The donor vector was constructed via Gibson assembly, and the final plasmid was confirmed by restriction digests and Sanger sequencing. Cas9- and T7 RNA polymerase-expressing NF54 parasites were transfected by preloading erythrocytes with the donor vector (Deitsch et al., 2001). Cultures were maintained in 500 nM aTc (Sigma-Aldrich 37919) and 2.5 μg/mL blasticidin (RPI Corp B12150-0.1). Successful transfection was confirmed by measuring luciferase expression. We were unable to introduce an epitope tag into the 3’ end of *abci3* using this aptamer approach, and thus could not confirm reduced protein expression levels by Western blot. Nonetheless, evidence of a conditional knockdown was obtained by documenting substantially reduced parasite growth upon removal of aTc.

#### Parasite growth assays

To assess the effect on parasite viability of knocking down ABCI3 expression, synchronous ring-stage parasites were cultured in the presence (50 nM) or absence of aTc, in triplicate in a 96-well U-bottom plate (Corning 62406-121). Luminescence was measured at 0, 72, and 120 h post-invasion using the Renilla-Glo(R) Luciferase Assay System (Promega, E2750) and the GloMax Discover Multimode Microplate Reader (Promega). Luminescence values were normalized to 200 nM CQ-treated samples and results were visualized on a scatter plot using Prism (version 9; GraphPad).

#### Compound susceptibility assays

Compound susceptibility of the cKD parasites was assessed as described above. Compounds were assayed using 2-fold dilutions with inhibition measured after 56 h. Parasite viability was determined by staining the parasites with SYBR Green and MitoTracker Deep Red followed by flow cytometry and IC_50_ analysis, as described above.

#### Immunofluorescence assays

Indirect Immunofluorescence assays (IFAs) were performed in suspension as described (Gnadig et al., 2020). Briefly, parasites were fixed in 4% (v/v) formaldehyde (Thermo Fisher Scientific) for 1 h at room temperature. This was followed by a second fixation step that supplemented the 4% formaldehyde solution with 1 mM cysteine and CaCl_2_ followed by an overnight incubation at 4°C. Cells were then permeabilized on ice using 0.05% Triton X-100 in 1×PBS for 5 min. Autofluorescence was quenched with 50 mM glycine for 10 min. After two washes in 1× PBS the cells were resuspended in 1% (w/v) bovine serum albumin (BSA) in 1×PBS blocking buffer and incubated with the appropriate dilution for each primary antibody used: 1:200 for rabbit or mouse anti-Flag (Genscript), rabbit anti-ERD2 (BEI Recourses), rabbit anti-BiP (kindly provided by Dr. Min Zhang), 1:50 for rat anti-Rab5B or Rab7 (kindly provided by Dr. Gordon Langsley), or 1:200 for anti-PfCRT antibodies. This was followed by incubation with the corresponding species-specific secondary antibodies (Alexa Fluor 488-, 594- or 647- conjugated goat anti mouse or rabbit antibodies; Thermo Fisher) diluted 1:2000 in 1% BSA in 1× PBS. Thin blood smears of stained RBCs were prepared on microscope slides and mounted with cover slips using Prolong Diamond Antifade Mount with DAPI (Thermo Fisher). Parasites were imaged using a Nikon Eclipse Ti-E wide-field microscope equipped with a sCMOS camera (Andor) and a Plan-apochromate oil immersion objective with 100× magnification (1.4 numerical aperture). A minimum of 27 Z stacks (0.2 μm step size) were photographed for each parasitized RBC. NIS-Elements imaging software (Version 5.02, Nikon) was used to control the microscope and camera as well as to deconvolve the images (using 25 iterations of the Richardson-Lucy algorithm for each image). ImageJ (Fiji) (version 2.0.0-rc-68/1.52 h) was used to crop the images, adjust brightness and intensity, overlay channels, and prepare montages.

#### Measurement of drug cellular accumulation using the inoculum effect

In the absence of radioactively labelled compounds, we measured drug cellular accumulation using the inoculum effect assay (Geary et al., 1990). Briefly, highly-synchronized 3D7-A10, ABCI3 ^3 copies^ (resistant to **1**) and ABCI3 ^L690I ed.^ (resistant to **4**) parasite lines were exposed to serially diluted MMV compounds or CQ at parasitemias ranging from 0.25% - 4%. The inoculum size equals the parasitemia × hematocrit (Geary et al., 1990) and ranged from 0.75 to 6. The measure of absolute drug potency was achieved by extrapolating the linear relationship between increasing inoculum size and IC_50_ to an inoculum size of zero, using the following equation: IC_50_ measured = IC_50_ absolute + (IC_50_ absolute × accumulation ratio × fractional volume of parasitized RBCs) (Geary et al., 1990). The drug accumulation ratio (CAR) is equal to the measured IC_50_ minus the absolute IC_50_) / (absolute IC_50_ × fractional volume of parasitized RBCs) (Bray et al., 1996). This ratio represents the amount of drug in the parasitized RBC pellet to the amount of drug in a similar volume of medium.

#### Immuno-electron microscopy

To immunolocalize HA-tagged ABCI3, *P*. *falciparum* cultures were fixed in 4% paraformaldehyde (Polysciences Inc.) in 100 mM PIPES/0.5 mM MgCl_2_, pH 7.2 for 1 h at 4°C. Samples were then embedded in 10% gelatin and infiltrated overnight at 4°C with 2.3 M sucrose/20% polyvinyl pyrrolidone in PIPES/MgCl_2_. Samples were trimmed, frozen in liquid nitrogen, and sectioned with a Leica Ultracut UCT7 cryo-ultramicrotome (Leica Microsystems). 50 nm sections were blocked with 5% fetal bovine serum (FBS)/5% normal goat serum for 30 min and subsequently incubated with rabbit anti-HA antibody (Sigma) at 1:100 for 1 h, followed by secondary anti-rabbit IgG antibody conjugated to 18 nm colloidal gold (Jackson ImmunoResearch Laboratories) for 1 h. Sections were stained with uranyl acetate and lead citrate, and viewed on a JEOL 1200 EX transmission electron microscope (JEOL USA) equipped with an AMT 8 megapixel digital camera and AMT Image Capture Engine V602 software (Advanced Microscopy Techniques). All labeling experiments were conducted in parallel, with controls omitting the primary antibody. These controls were consistently negative at the concentrations of colloidal gold-conjugated secondary antibodies used in these studies.

#### Detergent-based β-hematin inhibition assays

A solution containing water + 305.5 μM Nonidet P-40 (NP-40) + DMSO at a v/v ratio of 70% + 20% + 10%, respectively, was prepared and 100 μL added to all wells in columns 1-11 in a flat-bottomed 96-well plate. Working stocks of test compounds and controls were constituted to 10 mM, from which 20 μL of each was added to wells in the final column (column 12) together with distilled water (140 μL) and 305.5 μM NP-40 detergent (40 μL). This effectively lowered the final drug concentration to 1mM. Each compound (100 μL) was then serially diluted from columns 12 to 2 (column 1 served as a blank). A 25 mM hematin stock solution was prepared by sonicating hemin in 100% DMSO for 3 min and 178.8 μL of this solution was suspended in 20 mL acetate buffer (1 M, pH 4.8) and thoroughly mixed. The homogenous suspension (100 μL) was then added to all wells to give final hematin concentrations of 100 mM and a drug concentration of 0.5 mM in column 12. Plates were covered and incubated at 37°C for 5 h after which 32 μL of 50% pyridine solution (20% (v/v) H_2_O, 20% (v/v) acetone and 2 M HEPES buffer (pH 7.4) and 50% pyridine) was added to each well to give a final pyridine concentration of 5% (v/v). Acetone (60 μL) was then added to assist with hematin dispersion. The UV-vis absorbance of the plate wells was read at 405 nm on a SpectraMax P340 plate reader. The β-hematin inhibitory IC_50_ values for each compound were computed from the blank-corrected absorbance values at 405 nm using sigmoidal dose-response curve fitting analysis (Prism version 9, GraphPad).

#### Cellular heme fractionation assays

NF54 parasite susceptibility to compounds **1**, **3**, **4**, **5**, CQ and pyrimethamine was determined in 72 h assays as described above. IC_50_ values were used to initiate heme fractionation studies (Combrinck et al., 2013, 2015)). Briefly, ring-stage NF54 parasites were synchronized by treating them for two cycles using 5% sorbitol. The ∼3-5 h old parasites were then incubated in a gradient of IC_50_ concentrations (based on the 72 h chemosensitivity assay) ranging from 0.5-4× at 5% parasitemia and 2% hematocrit. A no-drug control was included. After 28 h, late trophozoites were harvested by lysing RBCs with 0.05% saponin followed by multiple washes with 1×PBS (pH 7.5) to remove traces of RBC Hb. Pellets were then resuspended in 1×PBS (pH 7.5) and an aliquot of the trophozoite suspension was stained with 1×SYBR Green and 100 nM MitoTracker Deep Red and quantified via flow cytometry to determine the total number of trophozoites. The remaining trophozoites were then released from their host RBCs by hypotonic lysis and sonication. The fractions corresponding to digested Hb, free heme and Hz were then carefully recovered through centrifugation and treatment with HEPES buffer (pH 7.4), 4% SDS, 25% pyridine solution, 0.3M HCl and 0.3M NaOH. The UV−visible spectrum of each heme fraction was measured as a Fe(III)heme−pyridine complex using a multi-well SpectraMax P340 plate reader. The total amount of each heme species was quantified using a heme standard curve where the mass of each heme-Fe species per trophozoite (fg/cell) was calculated by dividing the total amount of each heme species by the corresponding number of parasites in that fraction, as determined by flow cytometry. Statistical comparisons and analyses for trends were made using Prism (version 9, GraphPad) using Students’ t-tests with Welch corrections.

#### Binding studies with monomeric heme

A stock solution (1.2 mM) of hemin (Molecular weight 651.94 g/mole) was prepared in 100% DMSO. A 10 μM working solution was then prepared in 40% DMSO and 0.02 M HEPES buffer (pH 7.4) constituted in ultrapure HPLC-grade water, to maintain the heme in a strictly monomeric state. A 10 μM working solution of hemin at pH 5.6 was prepared in an identical manner except that HEPES was substituted with 0.02 M 2-[N-morpholino]ethanesulphonate (MES). Test compounds were diluted in 40% DMSO and 0.02 M HEPES buffer (pH 7.4) to obtain 2 mM working stocks while a 2 mM CQ diphosphate stock was also prepared in 40% DMSO and 0.02 M HEPES buffer (pH 7.4). All working solutions were covered in aluminum foil to avoid any photodegradation and used immediately upon preparation. Hemin solutions (10 μM, 2 mL) at the two pH values were titrated with increasing concentrations of the different compounds and absorbance values were recorded at 402 nm subsequent to each addition of a known volume of compounds. Data were corrected for dilution and analyzed using a standard non-linear least squares fitting method, assuming a 1:1 complexation model in which the change in absorbance (A) as a function of drug concentration in the hemin solution [D] is given by the equation below:

A = A_0_ + A∞K[D]/1 + K[D]

where A_0_ is the initial absorbance (no test sample), A∞ is the final limiting absorbance for the Fe(III)-drug complex, and K is the conditional association constant. Titrations were performed in triplicate and the average association constant (K) values reported with the standard error of the mean.

### Quantification and statistical analysis

Details regarding statistical tests are reported in the legends to Figures 2, 3, 4, 5, 6, and 7 and the supplemental information (Figures S4 and S5 and Tables S1–S7). We employed Mann-Whitney *U* tests throughout, except for the heme fractionation studies reported in Figure 6 and Table S6 that used two-tailed Student's tests (with Welch’s correction). Statistical analyses employed GraphPad Prism versions 8 or 9.

## Data Availability

All datasets generated during this study are provided in separate spreadsheets as part of Tables S1–S3. No code was generated. Any additional information required to reanalyze the data reported in this paper is available from the lead contact upon request.

## Supplemental information

Document S1. Figures S1–S5 and Tables S1–S10

Document S2. Article plus supplemental information

## Footnotes

Supplemental information can be found online at https://doi.org/10.1016/j.chembiol.2021.06.006.
