# Supplementary material for: The Plasmodium falciparum ABC transporter ABCI3 confers parasite strain-dependent pleiotropic antimalarial drug resistance
Source: Cell Chem Biol. 2022 May 19;29(5):824–839.e6. doi: 10.1016/j.chembiol.2021.06.006 (PMC8727639; doi:10.1016/j.chembiol.2021.06.006)
Supplement: Document S1. Figures S1–S5 and Tables S1–S10 [file mmc1.pdf]

Cell Chemical Biology, Volume *29*

Supplemental information

The *Plasmodium falciparum* ABC transporter ABCI3 confers parasite strain-dependent pleiotropic antimalarial drug resistance

James M. Murithi, Ioanna Deni, Charisse Flerida A. Pasaje, John Okombo, Jessica L. Bridgford, Nina F. Gnädig, Rachel L. Edwards, Tomas Yeo, Sachel Mok, Anna Y. Burkhard, Olivia Coburn-Flynn, Eva S. Istvan, Tomoyo Sakata-Kato, Maria G. Gomez-Lorenzo, Annie N. Cowell, Kathryn J. Wicht, Claire Le Manach, Gavreel F. Kalantarov, Sumanta Dey, Maëlle Duffey, Benoît Laleu, Amanda K. Lukens, Sabine Ottilie, Manu Vanaerschot, Ilya N. Trakht, Francisco-Javier Gamo, Dyann F. Wirth, Daniel E. Goldberg, Audrey R. Odom John, Kelly Chibale, Elizabeth A. Winzeler, Jacquin C. Niles, and David A. Fidock

Supplementary Materials for

The *Plasmodium falciparum* ABC Transporter ABCI3 Confers Parasite Strain-Dependent Pleiotropic Antimalarial Resistance

James M. Murithi, Ioanna Deni, Charisse F.A. Pasaje, John Okombo, Nina F. Gnädig, Rachel L. Edwards, Tomas Yeo, Jessica L. Bridgford, Sachel Mok, Anna Y. Burkhard, Olivia Coburn-Flynn, Eva S. Istvan, Tomoyo Sakata-Kato, Maria G. Gomez-Lorenzo, Annie N. Cowell, Kathryn J. Wicht, Claire Le Manach, Gavreel F. Kalantarov, Sumanta Dey, Benoît Laleu, Maëlle Duffey, Amanda K. Lukens, Sabine Ottilie, Manu Vanaerschot, Ilya N. Trakht, Francisco-Javier Gamo, Dyann F. Wirth, Daniel E. Goldberg, Audrey R. Odom John, Kelly Chibale, Elizabeth A. Winzeler, Jacquin C. Niles, David A. Fidock

Supplementary Figures and Tables

Figure S1. Genetic modifications of ABCI3 do not confer cross resistance to first-line antimalarials...**page 3**

Figure S2. ABCI3 mutations and amplifications do not show parasitemia-dependent dose responses against SNP-selecting compounds……………………………………………………………………………**page 4**

Figure S3. ABCI3 foci localize to vesicles and various cellular organelles……………………………...**page 5**

Figure S4. Parasites treated with compounds **3-5** do not display a heme fractionation profile similar to CQ ……………………………………………………………………………………………...**page 6**

Figure S5. PfMDR1 amplifications do not affect parasite susceptibility to ABCI3-associated compounds ……………………………………………………………………………………...**page 7**

Table S1. *Plasmodium falciparum* asexual blood stage IC_{50} data in nM for the tested antimalarials.**page 8**

Table S2. *Plasmodium falciparum* asexual blood stage IC_{50} data in nM for the antiplasmodial compounds assayed against the Dd2-B2 parent as well as the selected and edited ABCI3 F689C and S696Y parasite lines……………………………………………………………………………...**page 9**

Table S3. *Plasmodium falciparum* asexual blood stage IC_{50} data in nM for compounds **1**, **3** and **4** against 3D7-A10 parent, drug-selected, and gene-edited L690I parasite lines……………………**page 10**

Table S4. *Plasmodium falciparum* asexual blood stage IC_{50} data in nM for ABCI3-linked antiplasmodial inhibitors tested in the presence or absence of aTc in an ABCI3 conditional knockdown parasite line………………………………………………………………………………...……**page 11**

Table S5. Cellular accumulation ratio of chloroquine and compound **1** in *Plasmodium falciparum* asexual blood stage parasites…………………..………………………………………………….………………….**page 12**

Table S6. Mean ± SEM amount of hemoglobin, free heme and hemozoin in drug-treated parasites represented as percent proportion or absolute amount of heme iron per cell in fg/cell and the in vitro β-hematin inhibition assay IC_{50} data in µM for the tested antimalarials………………**page 13**

Table S7. *Plasmodium falciparum* asexual blood stage IC_{50} data in nM for the antiplasmodial compounds tested against PfCRT and PfMDR1 isoforms…………………………………………………**page 14**

Table S8. Transmission electron microscopy image scoring of *Plasmodium falciparum* asexual blood stage parasite subcellular localization of anti-HA stained ABCI3-3×HA…………………………......**page 15**

Table S9. Association constants with Fe(III)PPIX in 40% DMSO and either 0.02 M HEPES (pH 7.4) or MES (pH 5.6)……………………………………………………………………………….…………**page 16**

Table S10. Oligonucleotides used in this study……………………………………………………………**page 17**

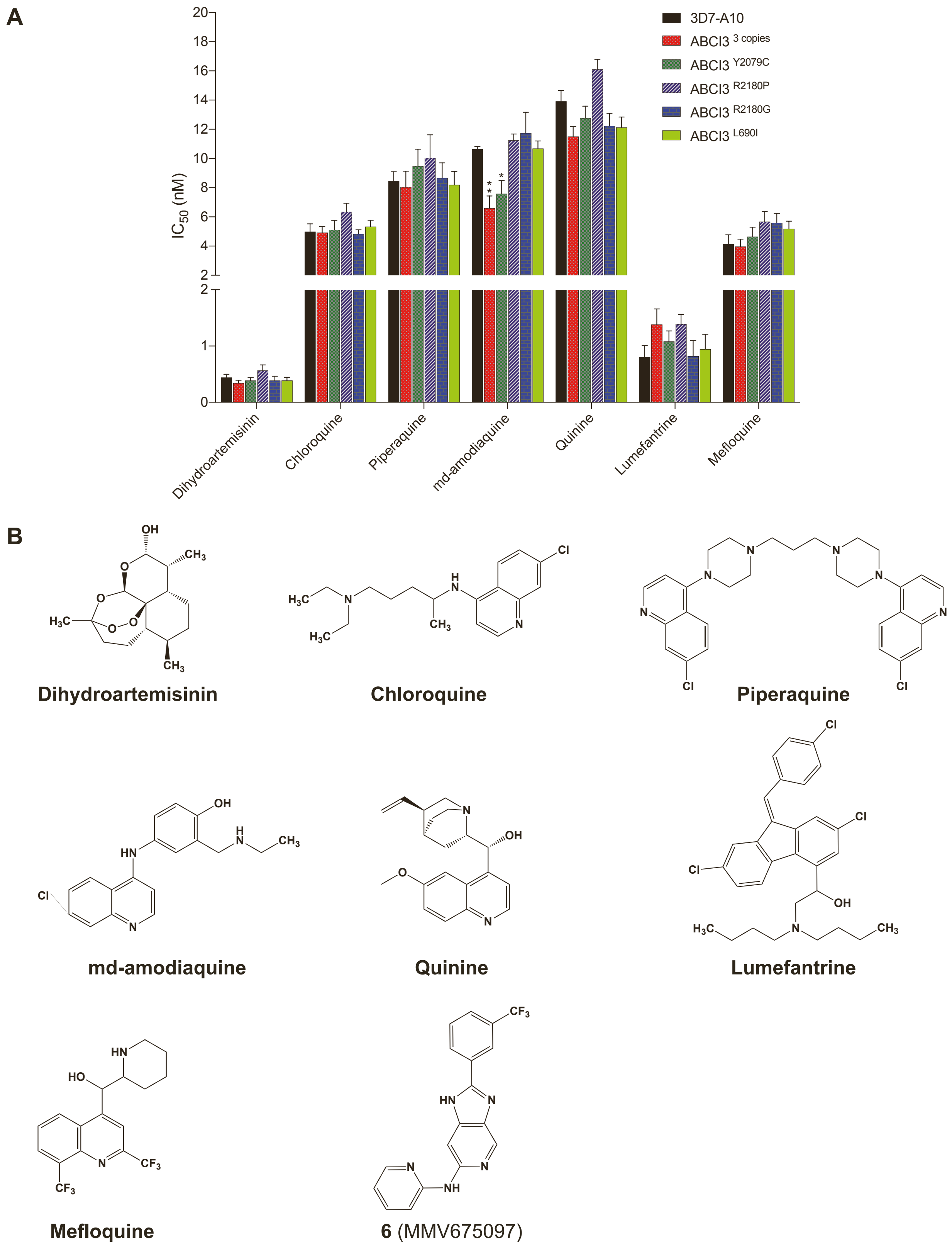

Figure S1 (related to Fig. 1, Table S1). Genetic modifications of ABCI3 do not confer cross resistance to first-line antimalarials. (**A**) Dose-response assays of ABCI3 CNV and SNP lines showed no cross-resistance against a panel of clinical antimalarials. Mean ± SEM; N≥4, n=2. Mann-Whitney *U* tests vs. 3D7-A10. (**B**) Chemical structure of first-line antimalarials and MMV compound **6**, which is identical to compound **1** apart from the absence of a –CF3 group in the pyridyl ring.

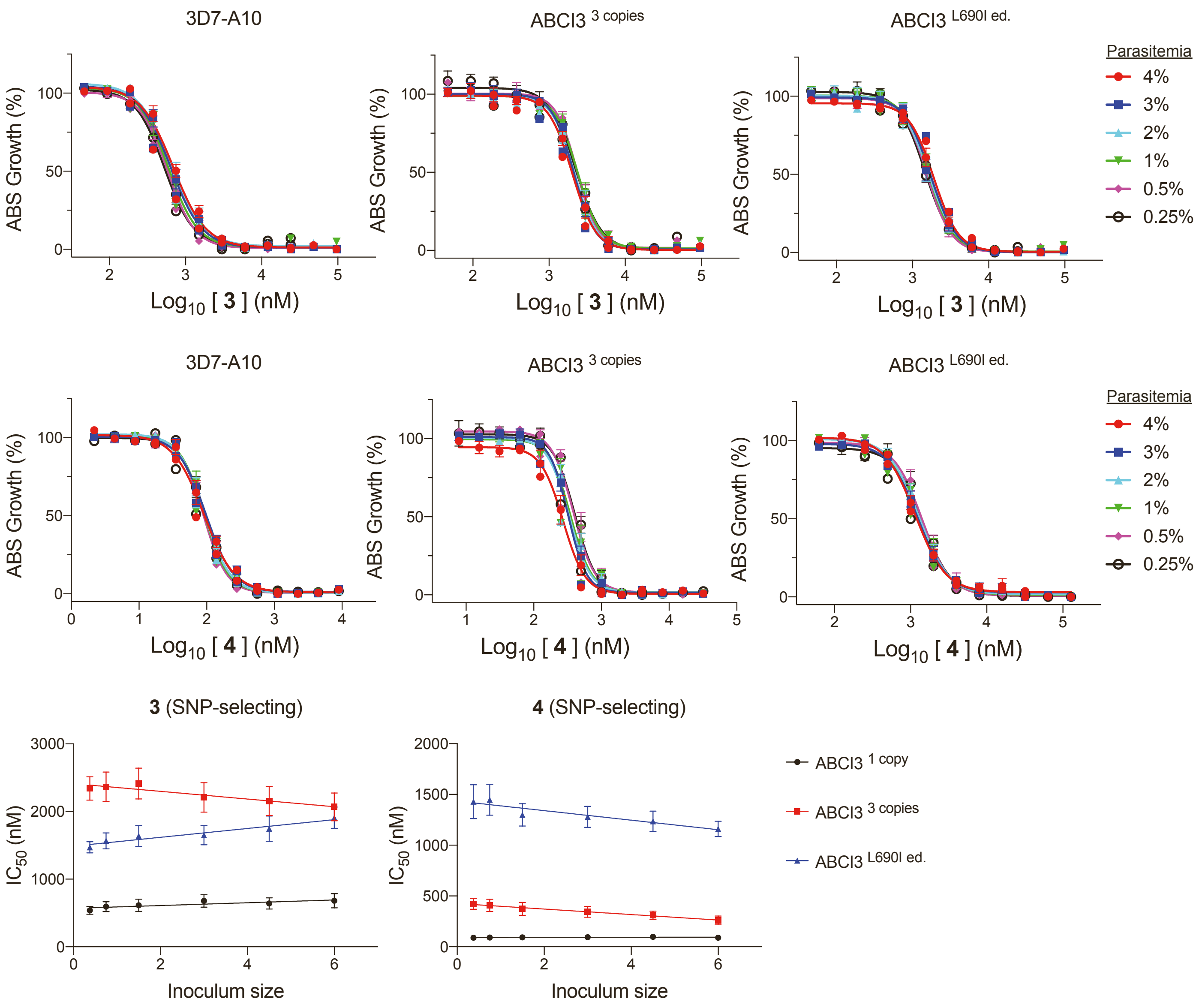

Figure S2 (related to Fig. 5). ABCI3 mutations and amplifications do not show parasitemia-dependent dose responses against SNP-selecting compounds. Compounds **3** and **4** had similar dose-response across all the three tested lines regardless of the starting parasite inoculum size. The absolute IC_{50} could therefore not be calculated from extrapolating the linear relationship between starting inoculum size and the measured IC_{50}. Mean ± SEM; N, n = 5, 2.

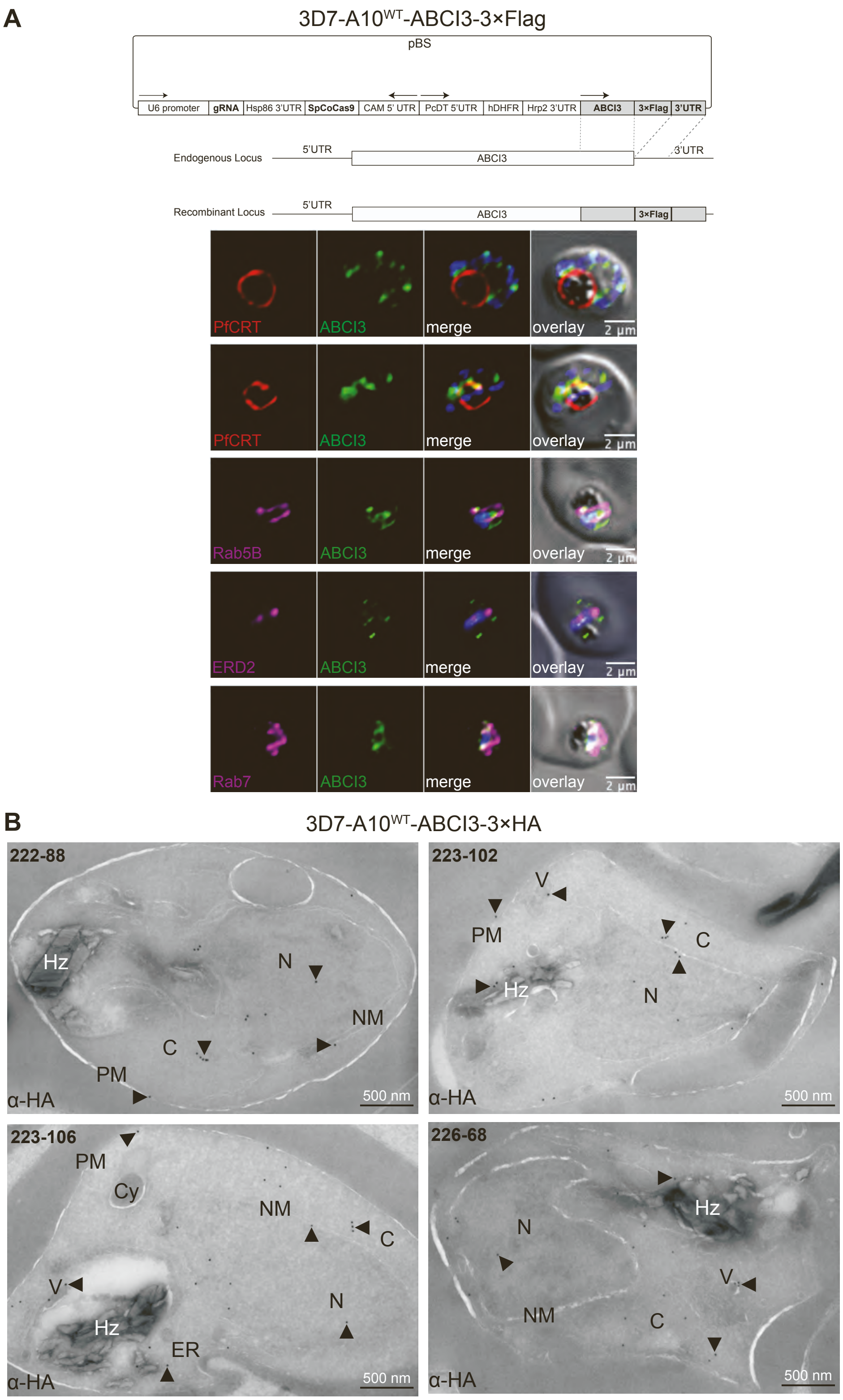

Figure S3 (related to Fig. 5D, E). ABCI3 foci localize to vesicles and various cellular organelles. (**A**) In the fluorescent image, ABCI3 Flag-tagged parasites were stained with anti-Flag (green), DAPI (nuclear, blue), anti-PfCRT (DV membrane, red), anti-ERD2 (cis-Golgi), or anti-Rab5B or anti-Rab7 (markers of vesicular transport) antibodies. The plasmid used to generate the tagged lines is illustrated. Scale bars: 2 μm. (**B**) Immuno-EM images of HA-tagged ABCI3 parasites stained with anti-HA antibodies. ER, endoplasmic reticulum; N, nucleus; NM, nuclear membrane; Hz, hemozoin crystals (digestive vacuole); V, vacuole; PM, plasma membrane and C, cytosol. Cy, cytostome. Scale bar: 500 nm.

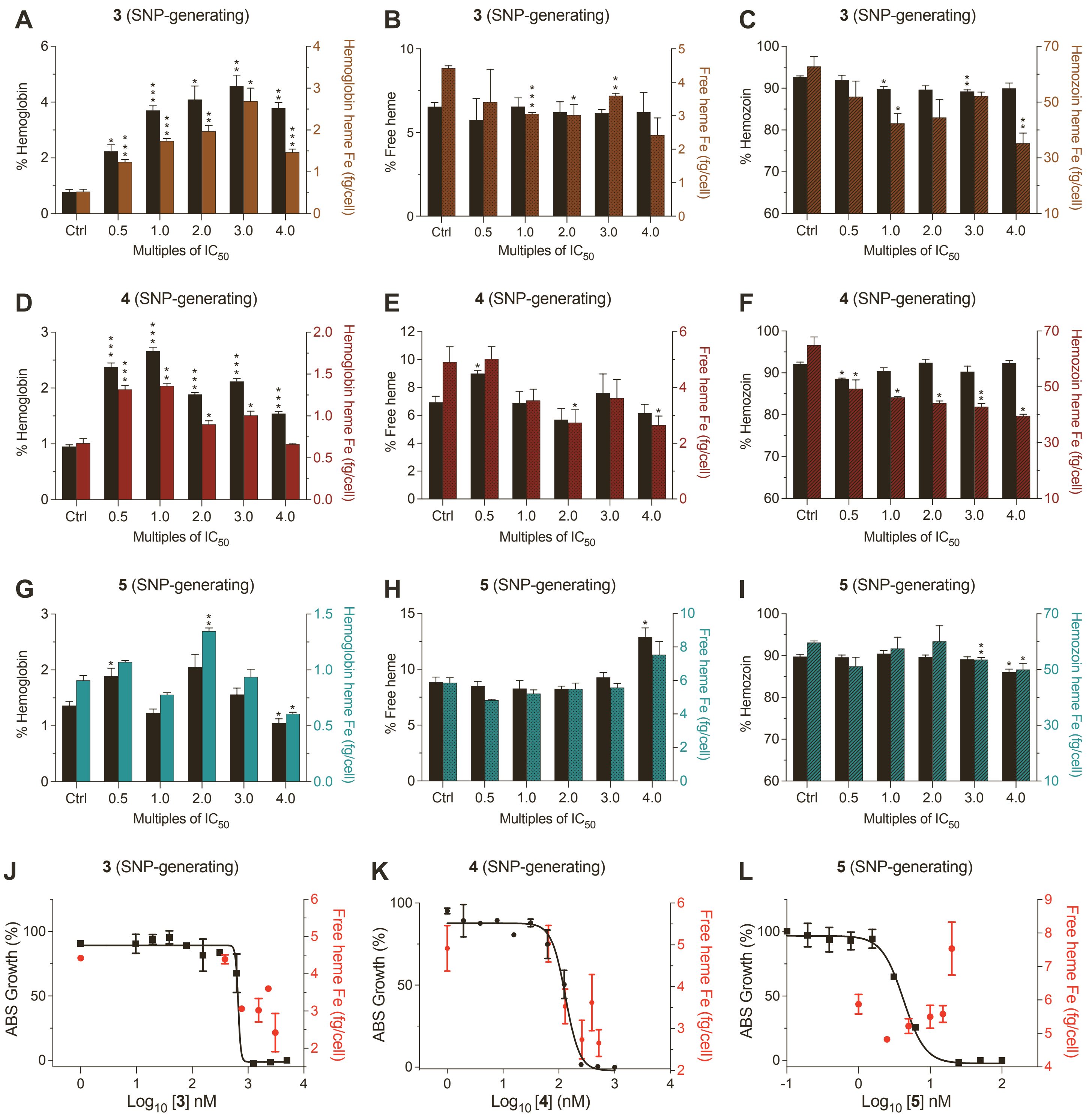

Figure S4 (related to Fig. 6). Parasites treated with compounds 3-5 do not display a heme fractionation profile similar to CQ. (**A-I**) Treatment of parasites with compounds **3-5** did not interfere with heme or Hz accumulation. (**J-L**) Concentration-dependent inhibition of parasite growth obtained with compounds **3-5** was independent of free heme levels. Statistical comparisons of the drug-treated lines to their untreated controls were performed using two-tailed Student's tests (with Welch's correction). N, n = 3, 2. *$p<0.05$, **$p<0.01$, ***$p<0.001$.

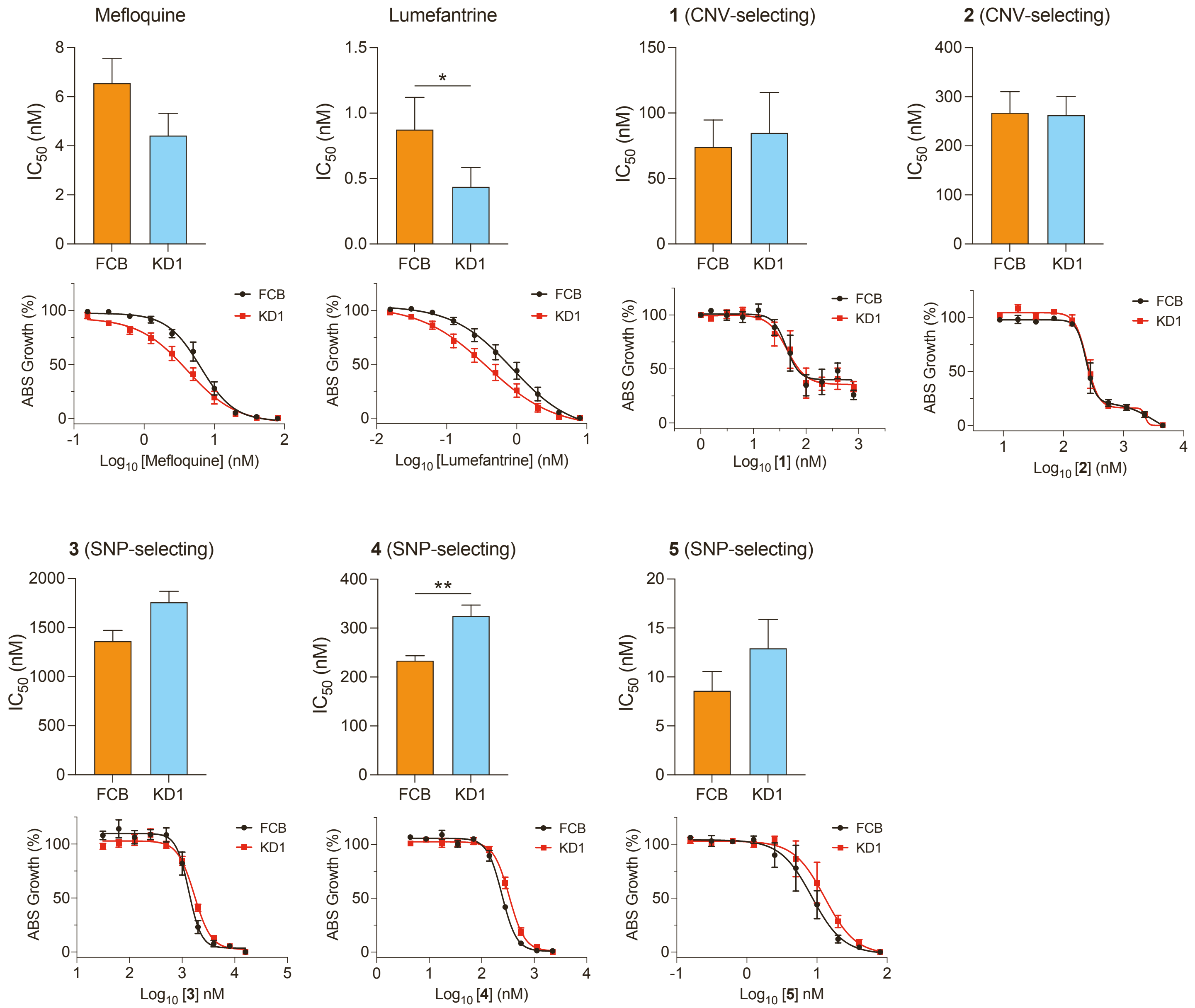

Figure S5 (related to Fig. 7, Table S7). PfMDR1 amplifications do not affect parasite susceptibility to ABCI3-associated compounds. Isogenic parasite lines expressing 1 (KD1) or 2 (FCB) copies of *pfmdr1* were equally susceptible to all five ABCI3-associated inhibitors. Mefloquine and lumefantrine were used as positive controls. *P* values were determined by comparison between the KD and parental FCB lines using Mann-Whitney *U* tests. N, n = 3-5, 2; *p<0.05, **p<0.01.

Table S1 (related to Fig. 3, S1). ***Plasmodium falciparum*** **asexual blood stage IC_{50} data in nM for the tested antimalarials.**

Antimalarials	3D7-A10			ABCI3 [3 copies]				ABCI3 [Y2079C]				ABCI3 [R2180P]				ABCI3 [R2180G]				ABCI3 [L690I]			
	Mean IC_{50}	SEM	N	Mean IC_{50}	SEM	N	*P* value	Mean IC_{50}	SEM	N	*P* value	Mean IC_{50}	SEM	N	*P* value	Mean IC_{50}	SEM	N	*P* value	Mean IC_{50}	SEM	N	*P* value
**1**	47.0	0.8	8	106 (1249)	8.0 (79.0)	15	<0.001 (<0.0001)	48.0	0.8	14	ns	58.0	2.0	14	<0.001	56.0	2.0	5	<0.01	45.0	1.0	7	ns
**2**	281	19.0	6	265 (4054)	34.0 (69.0)	4	ns (0.0095)	252	13.0	6	ns	275	12.0	6	ns	260	14.0	6	ns	208	11.0	6	<0.01
**3**	1012	64.0	11	2890	246	5	<0.001	2746	89.0	15	<0.0001	3029	141	15	<0.0001	2784	196	8	<0.0001	2511	225	8	<0.0001
**4**	140	14.0	10	500	47.0	15	<0.0001	1241	34.0	8	<0.0001	1918	61.0	7	<0.001	1268	55.0	4	<0.01	2300	217	5	<0.001
**5***	2.0	0.1	10	25.0	0.9	11	<0.0001	8.0	0.7	6	<0.001	29.0	1.7	6	<0.001	21.0	1.0	6	<0.001	2.0	0.3	9	ns
**6**	16.0	2.0	11	32.0	3.0	15	<0.001	20.0	1.0	10	ns	25.0	1.0	10	<0.01	23.0	1.0	8	<0.05	17.0	0.7	8	ns
Dihydroartemisinin	0.4	0.1	6	0.3	0.0	6	ns	0.4	0.1	6	ns	0.6	0.1	6	ns	0.4	0.1	6	ns	0.4	0.1	6	ns
Chloroquine	5.0	0.5	4	4.9	0.4	5	ns	5.1	0.6	5	ns	6.4	0.6	6	ns	4.8	0.3	5	ns	5.3	0.4	5	ns
Piperaquine	8.5	0.6	5	8.0	1.1	5	ns	9.5	1.2	5	ns	10.0	1.6	5	ns	8.7	1.0	5	ns	8.2	0.9	5	ns
md-amodiaquine	10.6	0.2	6	6.6	0.8	6	<0.01	7.6	0.9	6	<0.05	11.3	0.4	5	ns	11.7	1.4	4	ns	10.7	0.5	5	ns
Quinine	13.9	0.7	6	11.5	0.7	4	ns	12.8	0.8	5	ns	16.1	0.7	6	ns	12.2	0.8	4	ns	12.1	0.7	4	ns
Lumefantrine	0.8	0.2	4	1.4	0.3	4	ns	1.1	0.2	4	ns	1.4	0.2	5	ns	0.8	0.3	5	ns	0.9	0.3	5	ns
Mefloquine	4.2	0.6	5	4.0	0.5	5	ns	4.6	0.6	6	ns	5.7	0.7	6	ns	5.6	0.7	5	ns	5.2	0.5	5	ns

SEM: standard error of the mean; N: number of biological repeats (with technical duplicates); () refers to the IC_{50} and SEM of the second shift of the biphasic curve. * Selections with compound **5** were run on a Dd2-B2 parental background (IC_{50} = 8 nM). *P* values were determined by comparison between the variant lines and parental 3D7-A10 using Mann-Whitney *U* tests. ns: not significant (p>0.05).

Table S2 (related to Fig. 3). *Plasmodium falciparum* asexual blood stage IC_{50} data in nM for the antiplasmodial compounds assayed against the Dd2-B2 parent as well as the selected and edited ABCI3 F689C and S696Y parasite lines.

	Dd2-B2			ABCI3 $^{F689C\ ed.}$				ABCI3 $^{S696Y\ ed.}$			
Antimalarials	Mean IC_{50}	SEM	N	Mean IC_{50}	SEM	N	*P* value	Mean IC_{50}	SEM	N	*P* value
**1**	27.0 (1404)	3.0 (343)	4	30.0	3.0	6	--	34.0	2.0	6	--
**2**	265 (3542)	14.0 (465)	5	257	26.0	7	--	279	26.0	7	--
**3**	1546	95.0	7	956	112	7	<0.01	>5 mM		4	<0.01
**4**	246	20.0	7	36.0	4.0	7	<0.001	>10 mM		4	<0.01
**5***	8.0	0.9	6	94.0 (89.0)	10.0 (4.0)	6	<0.01	1626 (1433)	56.0 (24.0)	7	<0.01

SEM: standard error of the mean; N: number of biological repeats (with technical duplicates); For compounds **1** and **2**, the brackets denote the mean IC_{50} and SEM of the second shift of the biphasic curve. For compound **5** where selections were run on a Dd2-B2 parental background, the brackets denote the mean IC_{50} and SEM values of the selected clones. ABCI3 $^{F689C\ ed.}$/ ABCI3 $^{S696Y\ ed.}$: *P. falciparum* lines generated by introducing ABCI3 F689C and S696Y mutations into parental Dd2-B2 using CRISPR/Cas9. p values were determined by comparison between the variant lines and parental Dd2-B2 using Mann-Whitney *U* tests. -- not determined.

Table S3 (related to Fig. 2C). ***Plasmodium falciparum*** **asexual blood stage IC_{50} data in nM for compounds 1, 3 and 4 against 3D7-A10 parent, drug-selected, and gene-edited L690I parasite lines.**

	3D7-A10			ABCI3 L690I				ABCI3 L690I ed.			
Compounds	Mean IC_{50}	SEM	N	Mean IC_{50}	SEM	N	*P* value	Mean IC_{50}	SEM	N	*P* value
**1**	23.0	2.6	6	16.0	2.4	4	ns	16.0	0.8	4	ns
**3**	1548	161	4	3616	299	4	<0.05	3327	436	4	<0.05
**4**	188	30.0	4	4168	286	4	<0.05	3805	585	4	<0.05

SEM: standard error of the mean; N: number of biological repeats (with technical duplicates). ABCI3 L690I: *P. falciparum* line generated from selections with compound **4**. ABCI3 L690I ed.: *P. falciparum* line generated by introducing the ABCI3 L690I mutation into parental 3D7-A10 using CRISPR/Cas9. *P* values were determined by comparison between the variant lines and parental 3D7-A10 using Mann-Whitney *U* tests. ns: $p>0.05$.

Table S4 (related to Fig. 4). ***Plasmodium falciparum*** **asexual blood stage IC_{50} data in nM for ABCI3-linked antiplasmodial inhibitors tested in the presence or absence of aTc in an ABCI3 conditional knockdown parasite line.**

	50 nM aTc			3 nM aTc				0 nM aTc			
Antimalarials	Mean IC_{50}	SEM	N	Mean IC_{50}	SEM	N	*P* value	Mean IC_{50}	SEM	N	*P* value
Chloroquine	6.4	0.2	4	6.4	0.1	3	ns	6.0	0.4	4	ns
**1**	60.2	12.0	7	28.7	4.6	8	<0.05	19.4	3.4	7	<0.01
**2**	236	13.5	8	160	6.2	7	<0.001	128	8.7	8	<0.001
**3**	804	35.2	7	153	4.2	8	<0.001	74.9	9.6	7	<0.001
**4**	117	5.5	8	24.5	0.9	7	<0.001	14.1	0.3	8	<0.001
**5**	10.0	2.1	7	2.4	0.6	7	<0.01	1.4	0.4	8	<0.001
**6**	28.3	5.4	7	18.0	2.6	8	ns	12.9	2.7	7	<0.05

SEM: standard error of the mean; N: number of biological repeats (with technical duplicates). *P* values were determined by comparing the IC_{50} values of parasites cultured with 3 or 0 nM aTc with those cultured at 50 nM, using Mann-Whitney *U* tests. ns: p>0.05.

Table S5 (related to Fig. 5C). Cellular accumulation ratio of chloroquine and compound 1 in *Plasmodium falciparum* asexual blood stage parasites.

	3D7-A10			ABCI3 [3 copies]				ABCI3 [L690I ed.]			
Antimalarials	Mean IC_{50}	SEM	N	Mean IC_{50}	SEM	N	*P* value	Mean IC_{50}	SEM	N	*P* value
Chloroquine	36553	2385	6	17658	414	6	<0.01	28916	729	6	<0.01
**1**	59915	2678	6	2082	426	6	<0.01	25744	1288	6	<0.01

SEM: standard error of the mean; N: number of biological repeats (with technical duplicates). *P* values were determined by comparing accumulation levels between the variant lines and parental 3D7-A10, using Mann-Whitney *U* tests.

Table S6 (related to Fig. 6, S4): Mean ± SEM amount of hemoglobin, free heme and hemozoin in drug-treated parasites represented percent proportion or absolute amount of heme iron per cell in fg/cell and the *in vitro* β-hematin inhibition assay IC_{50} **data in μM for the tested antimalarials.**

Compound	Drug concentration	% Heme species			Heme Fe (fg/cell)			**β-hematin inhibition assay**		
		Hemoglobin	Free heme	Hemozoin	Hemoglobin	Free heme	Hemozoin	Mean IC_{50}	SEM	N
**Chloroquine**	No drug control	1.08 ± 0.11	6.12 ± 0.34	92.79 ± 0.31	0.80 ± 0.09	4.49 ± 0.14	68.30 ± 1.79	20.0	1.2	3
	0.5×IC_{50} (6 nM)	0.65 ± 0.14	6.72 ± 0.40	92.62 ± 0.32	0.47 ± 0.10	4.78 ± 0.19	66.04 ± 2.95			
	1.0×IC_{50} (12 nM)	0.65 ± 0.08*	7.63 ± 0.42	91.71 ± 0.38	0.47 ± 0.05*	5.48 ± 0.24*	65.91 ± 2.22			
	2.0×IC_{50} (24 nM)	1.12 ± 0.02	11.09 ± 0.35***	87.79 ± 0.35**	0.70 ± 0.01	6.92 ± 0.14**	54.83 ± 1.45*			
	3.0×IC_{50} (36 nM)	1.82 ± 0.03*	10.23 ± 0.28***	87.95 ± 0.24***	1.20 ± 0.06*	6.78 ± 0.31**	58.30 ± 2.51*			
	4.0×IC_{50} (48 nM)	0.78 ± 0.10	12.23 ± 0.41***	86.99 ± 0.32***	0.46 ± 0.06*	7.17 ± 0.27**	51.05 ± 1.15**			
**Pyrimethamine**	No drug control	2.12 ± 0.11	5.85 ± 0.28	92.04 ± 0.48	1.43 ± 0.13	3.94 ± 0.18	62.11 ± 0.54	>500	>500	3
	0.5×IC_{50} (12.5 nM	2.56 ± 0.14	5.53 ± 0.20	91.91 ± 0.29	1.61 ± 0.12	3.51 ± 0.21	58.13 ± 1.54			
	1.0×IC_{50} (25 nM)	2.61 ± 0.08	5.51 ± 0.21	91.88 ± 0.29	1.64 ± 0.16	3.58 ± 0.19	59.77 ± 3.21			
	2.0×IC_{50} (50 nM)	2.48 ± 0.14	5.47 ± 0.13	92.05 ± 0.26	1.57 ± 0.09	3.47 ± 0.13	58.27 ± 1.26*			
	3.0×IC_{50} (75 nM)	3.15 ± 0.12**	5.76 ± 0.13	91.09 ± 0.11	1.89 ± 0.11*	3.46 ± 0.11	54.62 ± 1.02**			
	4.0×IC_{50} (100 nM)	2.76 ± 0.08	5.53 ± 0.22	91.71 ± 0.27	1.72 ± 0.14	3.40 ± 0.13	56.49 ± 1.56*			
**1**	No drug control	3.12 ± 0.36	4.76 ± 0.50	92.12 ± 0.72	1.80 ± 0.21	2.73 ± 0.29	53.09 ± 0.32	29.3	2.2	3
	0.5×IC_{50} (24 nM)	3.81 ± 0.67	4.98 ± 0.43	91.21 ± 0.81	1.87 ± 0.37	2.84 ± 0.33	51.65 ± 0.74			
	1.0×IC_{50} (48 nM)	3.73 ± 0.48	5.45 ± 0.21	90.82 ± 0.54	1.84 ± 0.07	3.02 ± 0.21	50.28 ± 3.01			
	2.0×IC_{50} (96 nM)	3.48 ± 0.46	8.24 ± 0.31**	88.28 ± 0.45*	1.98 ± 0.28	4.67 ± 0.11*	50.18 ± 1.81			
	3.0×IC_{50} (144 nM)	3.71 ± 0.52	10.41 ± 0.18**	85.87 ± 0.64**	1.97 ± 0.31	5.48 ± 0.18**	45.17 ± 0.76***			
	4.0×IC_{50} (192 nM)	4.97 ± 0.83	13.32 ± 0.29***	81.71 ± 0.62***	2.83 ± 0.33	6.40 ± 0.29***	39.28 ± 1.55**			
**3**	No drug control	0.78 ± 0.09	6.55 ± 0.25	92.67 ± 0.27	0.53 ± 0.05	4.42 ± 0.07	62.83 ± 3.52	>500	>500	3
	0.5×IC_{50} (385 nM)	2.24 ± 0.24*	5.77 ± 1.27	92.00 ± 1.13	1.24 ± 0.06***	3.41 ± 0.98	52.01 ± 5.63			
	1.0×IC_{50} (770 nM)	3.07 ± 0.17***	6.55 ± 0.52	89.75 ± 0.68*	1.74 ± 0.06***	3.06 ± 0.04***	42.47 ± 3.47*			
	2.0×IC_{50} (1540 nM	4.09 ± 0.48*	6.22 ± 0.62	89.69 ± 0.88	1.97 ± 0.14**	3.02 ± 0.32*	44.52 ± 6.56			
	3.0×IC_{50} (2310 nM	4.57 ± 0.40**	6.16 ± 0.21	89.27 ± 0.34**	2.69 ± 0.31*	3.60 ± 0.07**	52.27 ± 1.42			
	4.0×IC_{50} (3080 nM	3.79 ± 0.20**	6.21 ± 1.17	90.0 ± 1.24	1.47 ± 0.08***	2.42 ± 0.51	35.27 ± 3.72**			
**4**	No drug control	0.95 ± 0.03	6.94 ± 0.44	92.11 ± 0.46	0.67 ± 0.05	4.92 ± 0.54	64.91 ± 2.97	>500	>500	3
	0.5×IC_{50} (65 nM)	2.38 ± 0.07***	9.00 ± 0.22*	88.62 ± 0.14*	1.32 ± 0.05***	5.03 ± 0.44	49.34 ± 3.13*			
	1.0×IC_{50} (130 nM)	2.66 ± 0.07***	6.91 ± 0.80	90.43 ± 0.78	1.36 ± 0.03**	3.53 ± 0.41	46.21 ± 0.37*			
	2.0×IC_{50} (260 nM)	1.89 ± 0.03***	5.70 ± 0.79	92.42 ± 0.82	0.90 ± 0.04*	2.74 ± 0.46*	44.10 ± 0.82*			
	3.0×IC_{50} (390 nM)	2.12 ± 0.05***	7.60 ± 1.37	90.28 ± 1.35	1.01 ± 0.05*	3.62 ± 0.67	42.89 ± 1.14**			
	4.0×IC_{50} (520 nM)	1.54 ± 0.04***	6.16 ± 0.63	92.29 ± 0.61	0.66 ± 0.01	2.65 ± 0.32*	39.60 ± 0.59*			
**5**	No drug control	1.36 ± 0.07	8.84 ± 0.47	89.80 ± 0.50	0.91 ± 0.04	5.87 ± 0.29	59.68 ± 0.57	>500	>500	3
	0.5×IC_{50} (2.5 nM)	1.89 ± 0.14*	8.50 ± 0.42	89.61 ± 0.56	1.07 ± 0.01	4.82 ± 0.06	51.16 ± 3.26			
	1.0×IC_{50} (5 nM)	1.23 ± 0.07	8.29 ± 0.71	90.48 ± 0.77	0.78 ± 0.02	5.22 ± 0.22	57.60 ± 4.04			
	2.0×IC_{50} (10 nM)	2.05 ± 0.22	8.26 ± 0.24	89.69 ± 0.46	1.35 ± 0.03**	5.50 ± 0.34	60.09 ± 5.64			
	3.0×IC_{50} (15 nM)	1.56 ± 0.12	9.29 ± 0.44	89.15 ± 0.55	0.94 ± 0.07	5.58 ± 0.25	53.59 ± 0.76**			
	4.0×IC_{50} (20 nM)	1.05 ± 0.07*	12.90 ± 0.81*	86.04 ± 0.75*	0.61 ± 0.01*	7.54 ± 0.79	50.00 ± 2.10*			

Mean±SEM amount of hemoglobin, free heme and hemozoin represented as percent and fg/cell. The amounts of heme in different parasite lines were determined by the heme fractionation assay (see methods). Parasites were treated with increasing concentrations of chloroquine, pyrimethamine or compounds **1,3-5** at different multiples of their IC_{50} values. Hemoglobin, free heme and hemozoin amounts were measured 30 h later. N, n = 1, >3. Statistical comparisons of the drug-treated lines to their untreated controls were performed using two-tailed Student's tests (with Welch's correction). *p<0.05; **p<0.01; ***p<0.001. Amodiaquine and doxycycline were also tested in biological triplicate in the *in vitro* β-hematin inhibition assays. The mean IC_{50} in μM and SEM were 9.4 and 1.3, respectively for amodiaquine and >500 and >500, respectively for doxycycline. Compound 2 was not tested because of lack of material.

Table S7 (related to Fig. 7, S5). ***Plasmodium falciparum*** **asexual blood stage IC_{50} data in nM for the antiplasmodial compounds tested against PfCRT and PfMDR1 isoforms.**

	PfCRT isoforms							PfMDR1 isoforms						
	Dd2 [3D7]			Dd2 [Dd2]				FCB			KD1			
Antimalarials	Mean IC_{50}	SEM	N	Mean IC_{50}	SEM	N	*P* value	Mean IC_{50}	SEM	N	Mean IC_{50}	SEM	N	*P* value
**1**	47.0	6.7	4	29.0(1234)	5.8(162)	4	ns (0.0286)	74.12	20.55	3	84.86	30.87	3	--
**2**	203	24.0	4	236(2780)	23.0(504)	4	ns (0.0286)	278.4	34.92	4	270	33.05	4	ns
**3**	563	18.0	4	1268	58.0	4	<0.05	1361	108.8	5	1760	111	5	ns
**4**	100	4.9	4	236	23.0	4	<0.05	233.5	10.05	5	324.8	22.4	5	<0.01
**5**	2.2	0.2	4	4.9	0.4	4	<0.05	8.59	1.96	4	12.93	2.94	4	ns
Chloroquine	11.0	2.2	4	101	12	4	<0.05	--			--			
Mefloquine	--			--				6.55	1.00	4	4.42	0.91	4	ns
Lumefantrine	--			--				0.64	0.10	4	0.29	0.04999	4	<0.05

SEM: standard error of the mean; N: number of biological repeats (with technical duplicates); () refers to the IC_{50} and SEM of the second shift of the biphasic curve. *P* values were determined by comparing the IC_{50} shift of the Dd2 [Dd2] parasite line with Dd2 [3D7] (PfCRT), or the FCB *pfmdr1* two-copy parasite line with its isogenic *pfmdr1* single-copy KD1 (PfMDR1) using Mann-Whitney *U* tests. ns: p>0.05. '--: not determined.

Table S8 (related to Fig. 5, S3B). Transmission electron microscopy image scoring of *Plasmodium falciparum* asexual blood stage parasite subcellular localization of anti-HA stained ABCI3-3×HA.

Sample number	Image number	Plasma membrane	Edoplasmic reticulum	Digestive vacuole	Vesicles	Nucleus	Nuclear membrane	Cytosol	Total ABCI3-3×HA label per sample
222	79	2	0	4	1	1	0	5	13
222	81	2	ND	1	ND	2	1	6	12
222	82	0	ND	1	0	2	0	1	4
222	83	1	0	1	0	4	0	3	9
222	85	3	ND	0	5	2	0	15	26
222	86	0	0	1	2	0	1	10	15
222	88	1	ND	0	0	2	7	6	17
222	89	1	2	2	0	5	0	4	15
222	90	0	3	0	0	5	3	2	13
222	91	1	3	0	1	0	2	5	13
223	95	0	ND	0	ND	2	1	6	9
223	96	0	ND	2	ND	3	0	8	13
223	97	0	ND	0	ND	2	1	4	7
223	99	1	ND	0	1	2	0	8	12
223	100	0	ND	2	0	4	1	3	10
223	102	1	0	3	1	4	1	8	18
223	104	2	2	0	ND	2	0	5	11
223	106	3	1	1	2	5	2	9	23
226	64	0	ND	0	0	0	0	3	3
226	65	0	0	0	0	1	3	7	11
226	66	0	2	1	0	3	0	4	11
226	68	0	0	2	3	3	1	11	20
226	70	0	ND	5	0	2	0	16	23
226	72	1	1	0	0	1	0	14	18
226	73	2	0	1	0	0	0	1	5
226	74	0	1	1	0	3	1	2	8
226	75	0	ND	0	0	0	1	8	9
226	77	1	0	0	1	2	0	4	8
226	78	0	7	3	4	1	1	2	19
226	79	1	ND	0	0	1	1	10	13
226	80	2	0	2	0	1	4	6	15
Total ABCI3-3×HA label per organelle		25	22	33	21	65	32	196	403
% ABCI3-3×HA label per organelle		**6**	**5**	**8**	**5**	**16**	**8**	**49**	

Results were collated from parasites obtained on three separate occasions for electron microscopy processing and imaging. ND: not determined.

Table S9 (related to Fig. 6). Association constants with Fe(III)PPIX in 40% DMSO and either 0.02 M HEPES (pH 7.4) or MES (pH 5.6).

	log *K*	
Compound	pH 5.6	pH 7.4
Chloroquine	5.16 ± 0.03	5.32 ± 0.03
**1**	3.64 ± 0.03	4.00 ± 0.08
**3**	2.54 ± 0.02	2.82 ± 0.03
**4**	2.53 ± 0.11	2.59 ± 0.02
**5**	2.58 ± 0.02	2.42 ± 0.04

Higher log *K* values indicate higher heme-binding constants, with chloroquine showing strong binding, **1** being intermediate and **3-5** yielding low values. Mean±SEM.

Table S10 (Related to STAR Methods). Oligonucleotides used in this study.

Experiment	Nucleotide Sequence (5' to 3')	Description	Lab name
L690I validation in 3D7-A10 (related to **Fig. 2C**)	GGGAAATAACTATGGAATATAAAAAAACAG	ABCI3 L690I donor fragment fwd	p6417
	GTTGTGTCGAAGAGGTATCATGGG	ABCI3 L690I donor fragment rev	p6418
	GTTTCGATATAAATAAAGAG	ABCI3 L690I guide RNA	p6387/p6388
F689C and S696Y validation in Dd2-B2 (related to **Fig. S1A**)	GACAAACAAAATGACGAATG	ABCI3 F689C guide RNA (1)	p8159/p8160
	GTTTCGATATAAATAAAGAG	ABCI3 F689C guide RNA (2)	p8165/p8166
	GAGGTACCGAGCTCGaattcCAGATGAAAAGGAGTATCAGG	ABCI3 F689C/S696Y donor fragment fwd (In-Fusion)	p8161
	GAAAAGTGCCACCTGacgtcCAATCCTTAAACACATTTGAC	ABCI3 F689C/S696Y donor fragment rev (In-Fusion)	p8162
cKD in NF54^{pCRISPR} line (related to **Fig. S3**)	GTACGGTACAAACCCGGAATTCGAGCTCGGAGAAATTGCTTTAAT GAGTTACATGGG	ABCI3 RHR forward	--
	GGGTATTAGACCTAGGGATAACAGGGTAATGGAAAAATATAAAAA ATGAAACTACACC	ABCI3 RHR reverse	--
	GTTTAACGACAAAGATATCG	sgRNA target site	--
ABCI3 3×Flag and 3×HA tagging in 3D7-A10 (related to **Fig. S5**)	ATTGCTTTAATGAGTTACAT	ABCI3 3' tagging guide RNA	p7421/p7422
	AGAGGTACCGAGCTCGaattcCTCATCTCACCAGAAGATATG	ABCI3 3' donor fragment fwd	p7423
	CGAAAAGTGCCACCTGacgtcTCTACAACTATATAAGAAACTCC	ABCI3 3' donor fragment rev	p7424
	GCAGAAAATTTATATTTTCAAAGTGGAGATTATAAAGATCATGATG GAGATTATAAAGATCATGATATAGATTATAAAGATGATGATGATAA Ataa	TEV + 3×Flag tag fragment	--
	TACCCATACGATGTTCCTGACTATGCTGGTTATCCTTATGACGTGC CTGACTATGCAGGATCCTATCCATATGACGTTCCAGATTACGCT	3×HA tag fragment	--

--: No oligonucleotides
